# Supplementary material for: Design, Green Synthesis and Tailoring of Vitamin E TPGS Augmented Niosomal Nano-Carrier of Pyrazolopyrimidines as Potential Anti-Liver and Breast Cancer Agents with Accentuated Oral Bioavailability
Source: Pharmaceuticals (Basel). 2022 Mar 9;15(3):330. doi: 10.3390/ph15030330 (PMC8949375; doi:10.3390/ph15030330)
Supplement: Supplementary file 1 [file pharmaceuticals-15-00330-s001.zip › pharmaceuticals-1604664-supplementary.pdf]

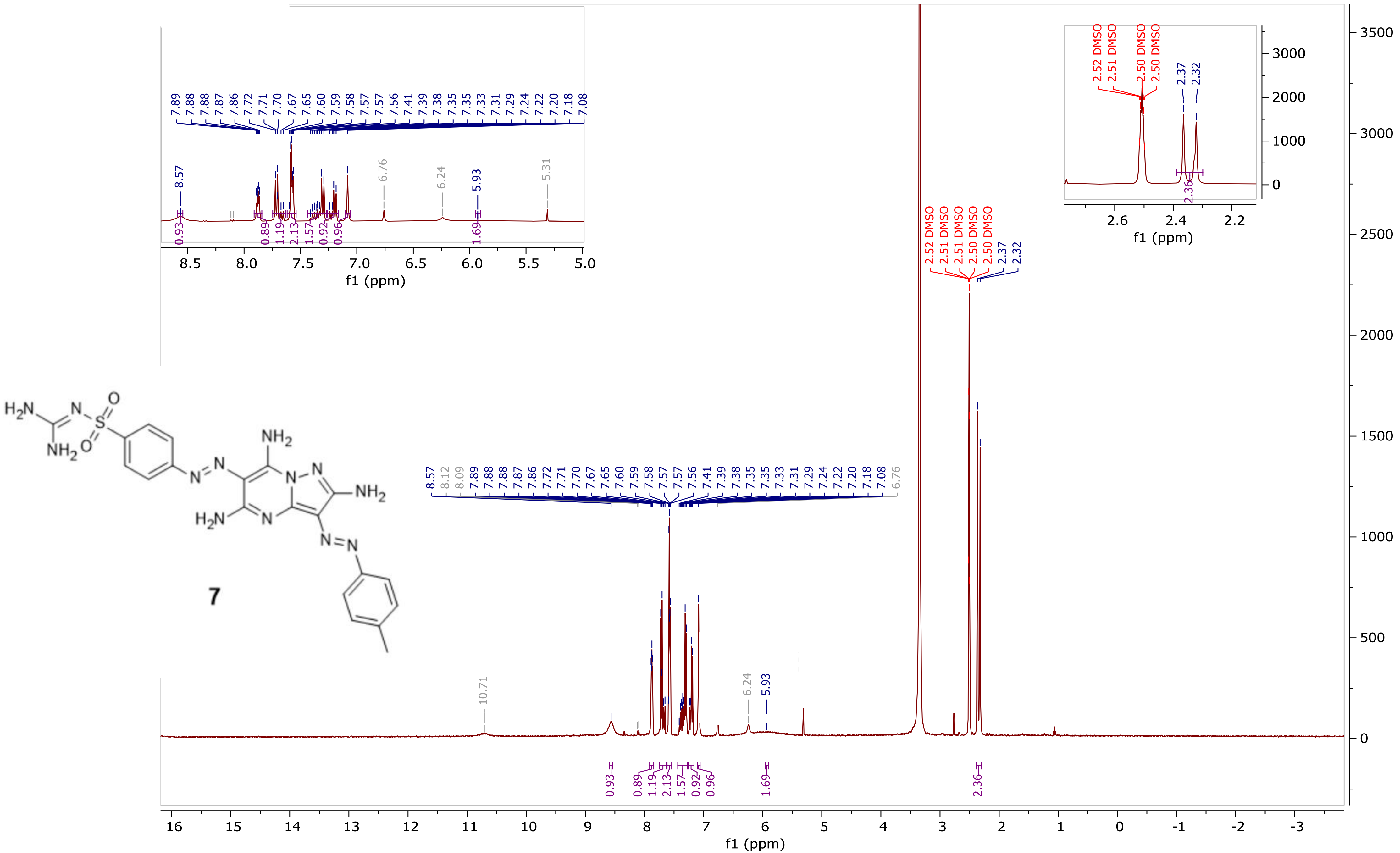

^{1}H-NMR spectrum of compound 7

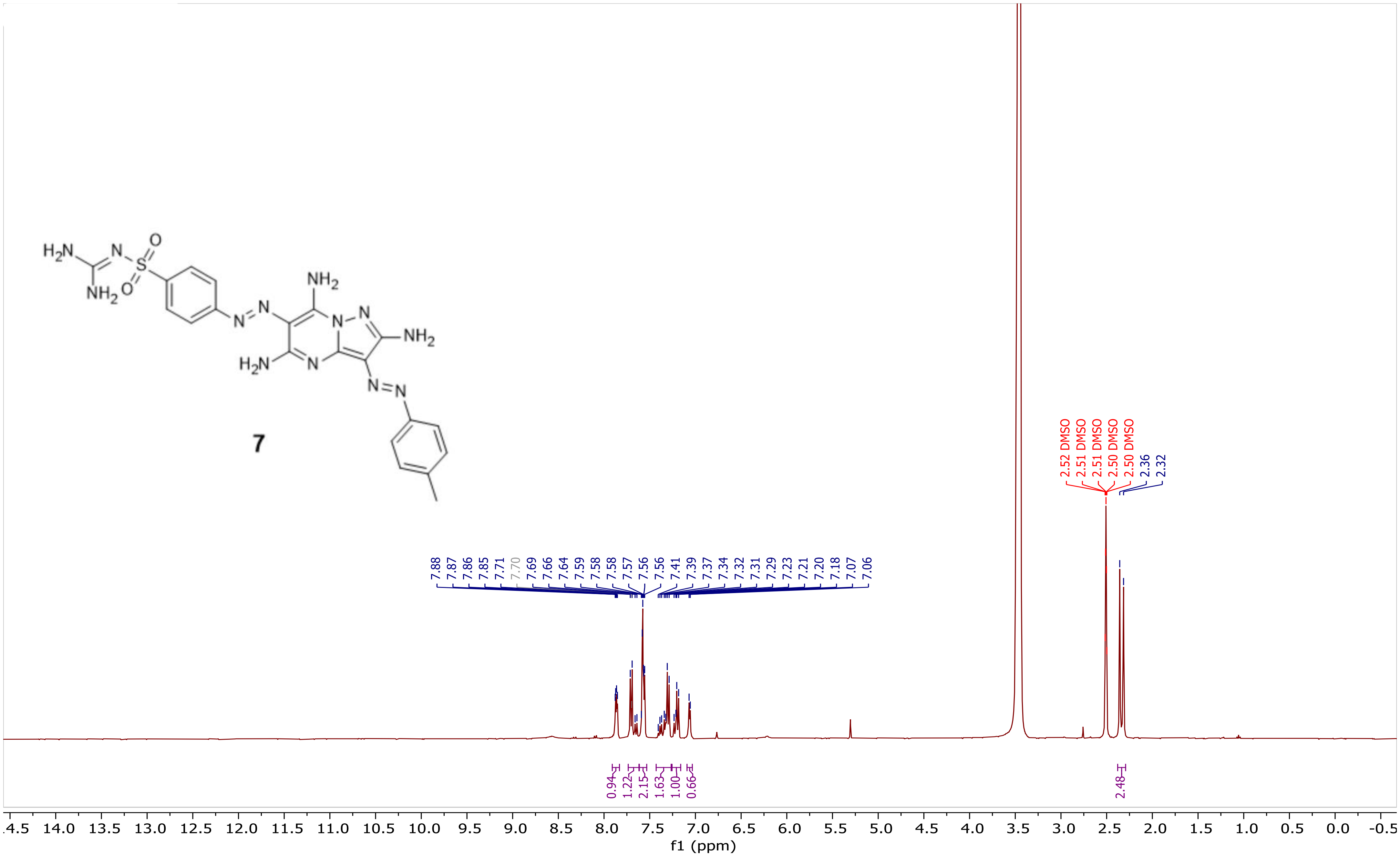

^{1}H-NMR – D_2O spectrum of compound 7

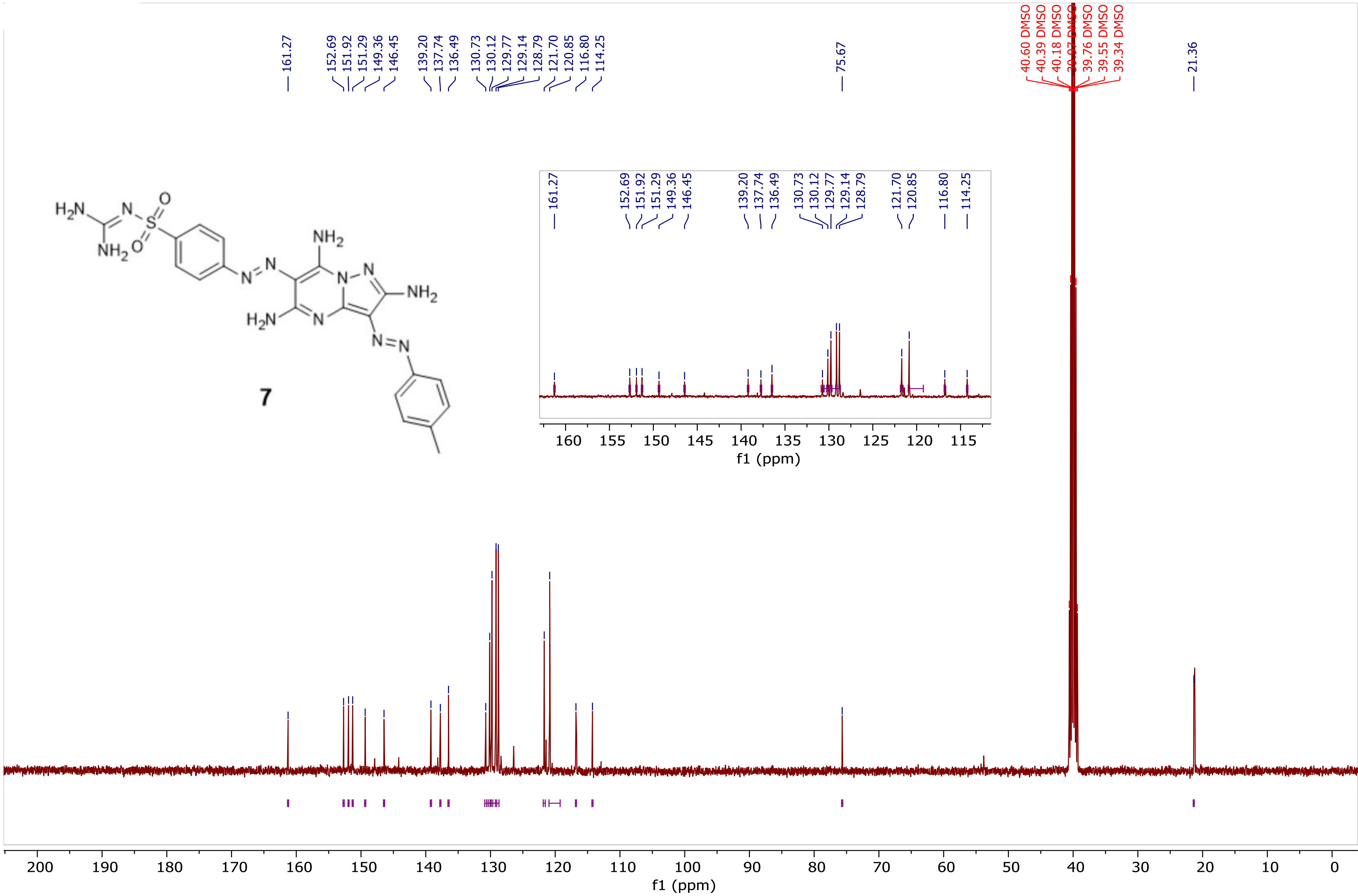

^{13}C-NMR spectrum of compound 7

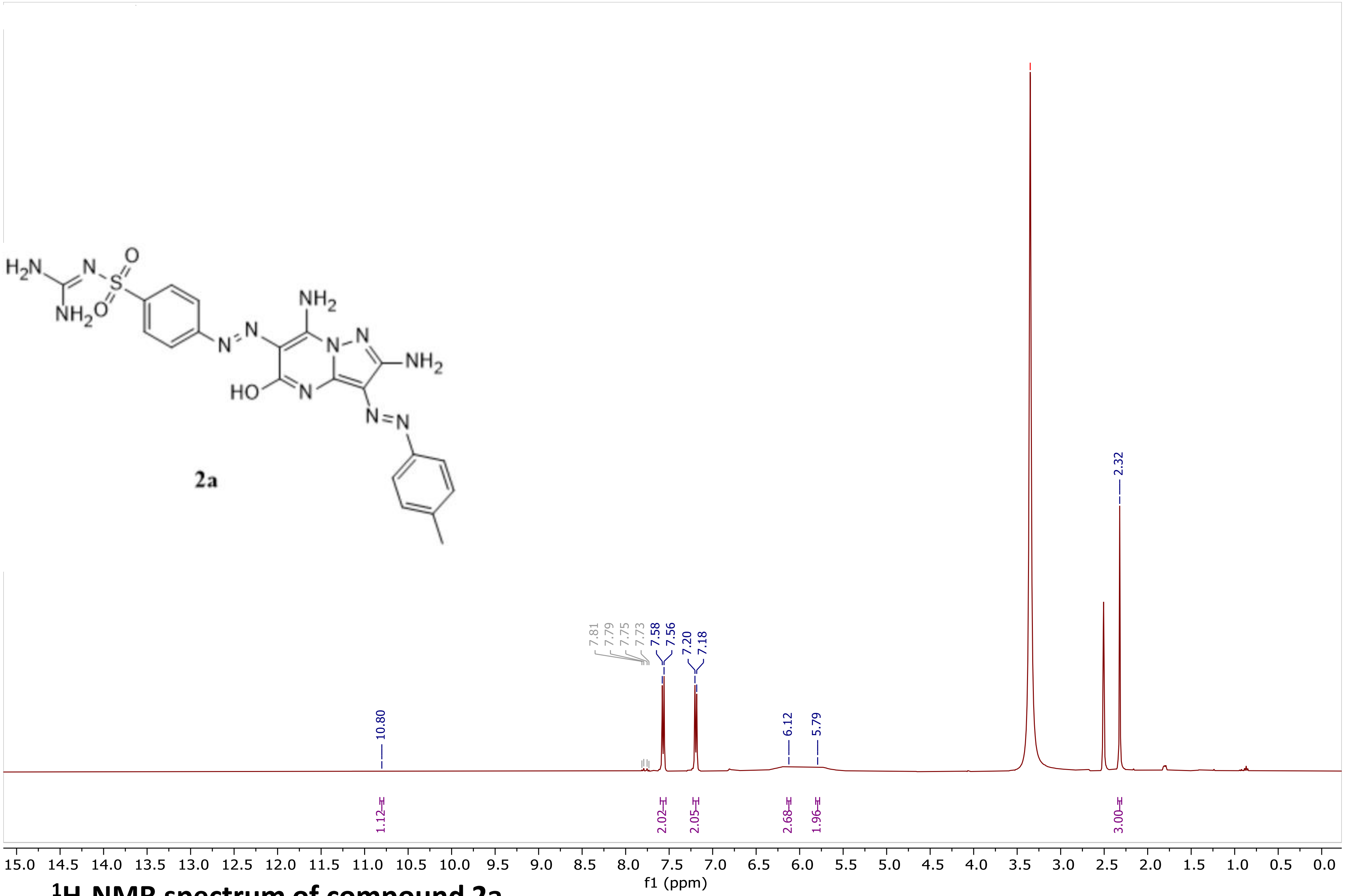

^{1}H-NMR spectrum of compound 2a

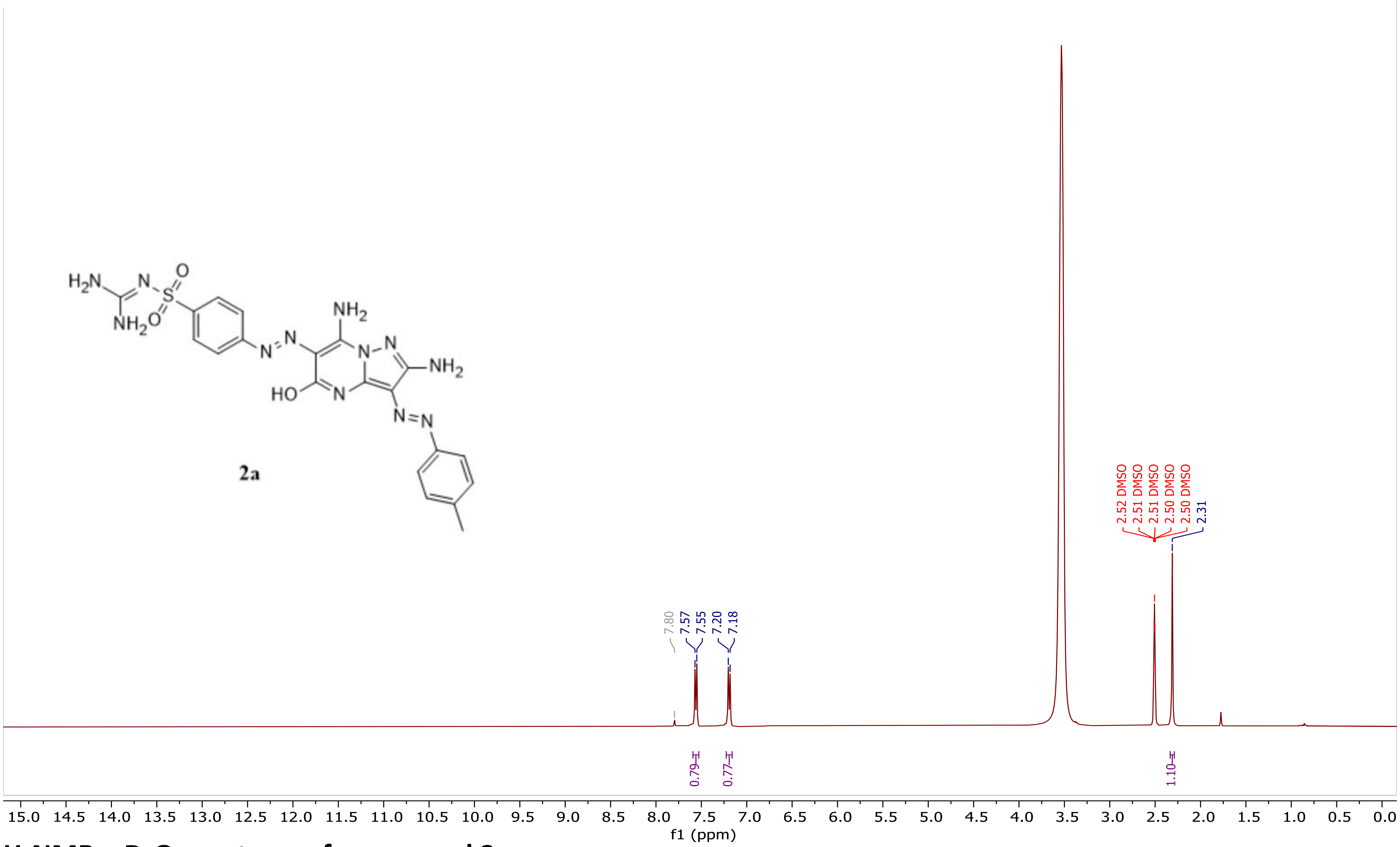

^{1}H-NMR – D_2O spectrum of compound 2a

IR spectrum of compound 2a

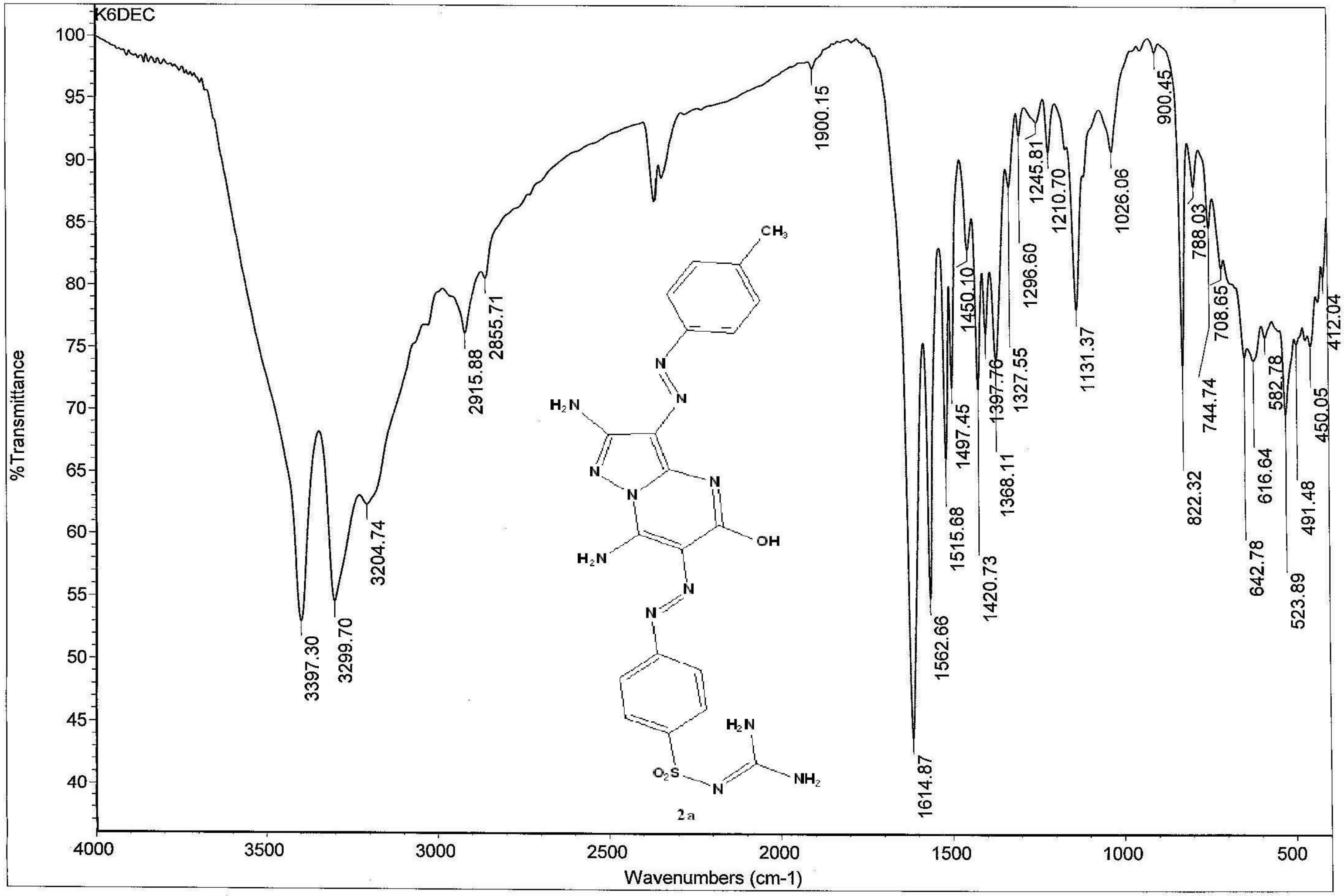

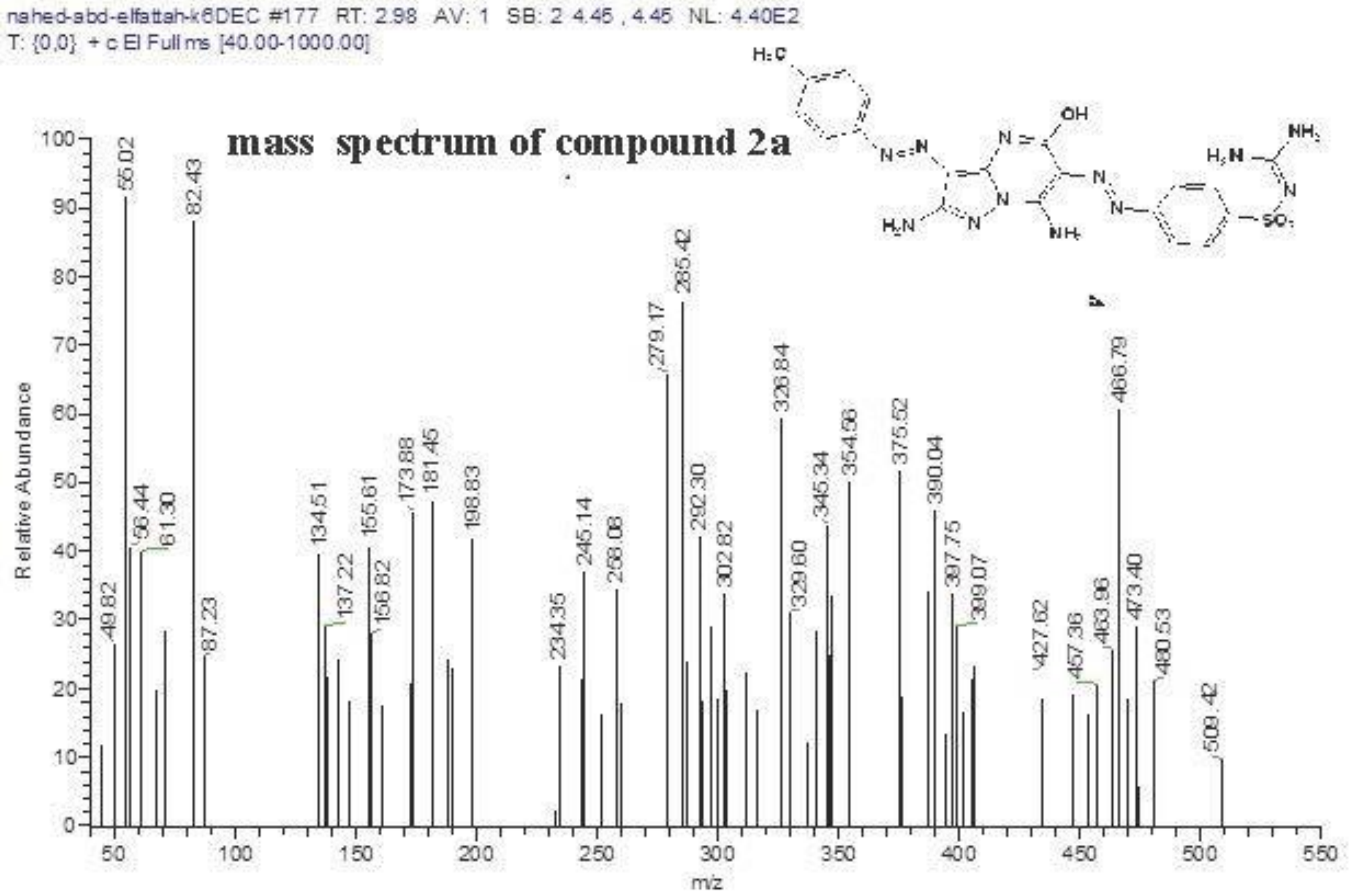
nahed-abd-elfattah-k6DEC #177 RT: 2.98 AV: 1 SB: 2 4.45 , 4.45 NL: 4.40E2
T: {0,0} + c EI Full ms [40.00-1000.00]
mass spectrum of compound 2a
Relative Abundance
m/z
55.02
82.43
56.44
61.30
49.82
87.23
134.51
137.22
155.61
156.82
173.88
181.45
198.83
234.35
245.14
258.08
279.17
285.42
292.30
302.82
326.84
329.60
345.34
354.56
375.52
390.04
397.75
399.07
427.62
457.36
463.96
466.79
473.40
480.53
509.42

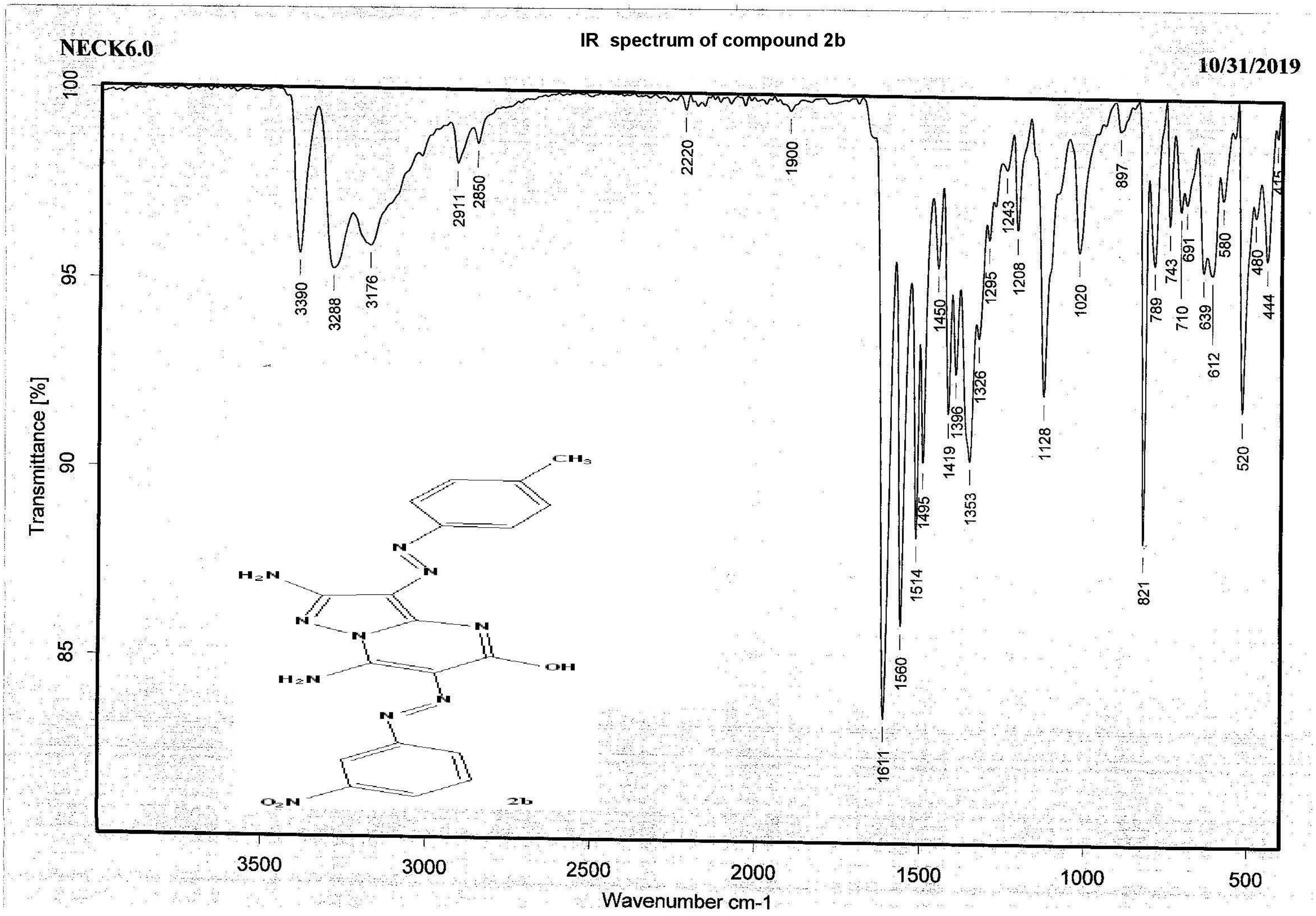

NECK6.0
IR spectrum of compound 2b
10/31/2019
Transmittance [%]
Wavenumber cm-1
100
95
90
85
3500
3000
2500
2000
1500
1000
500
3390
3288
3176
2911
2850
2220
1900
1611
1560
1514
1495
1450
1419
1396
1353
1326
1295
1243
1208
1128
1020
897
821
789
743
710
691
639
612
580
520
480
444
CH3
H2N
N
OH
O2N
2b

1H-NMR spectrum of compound 2b

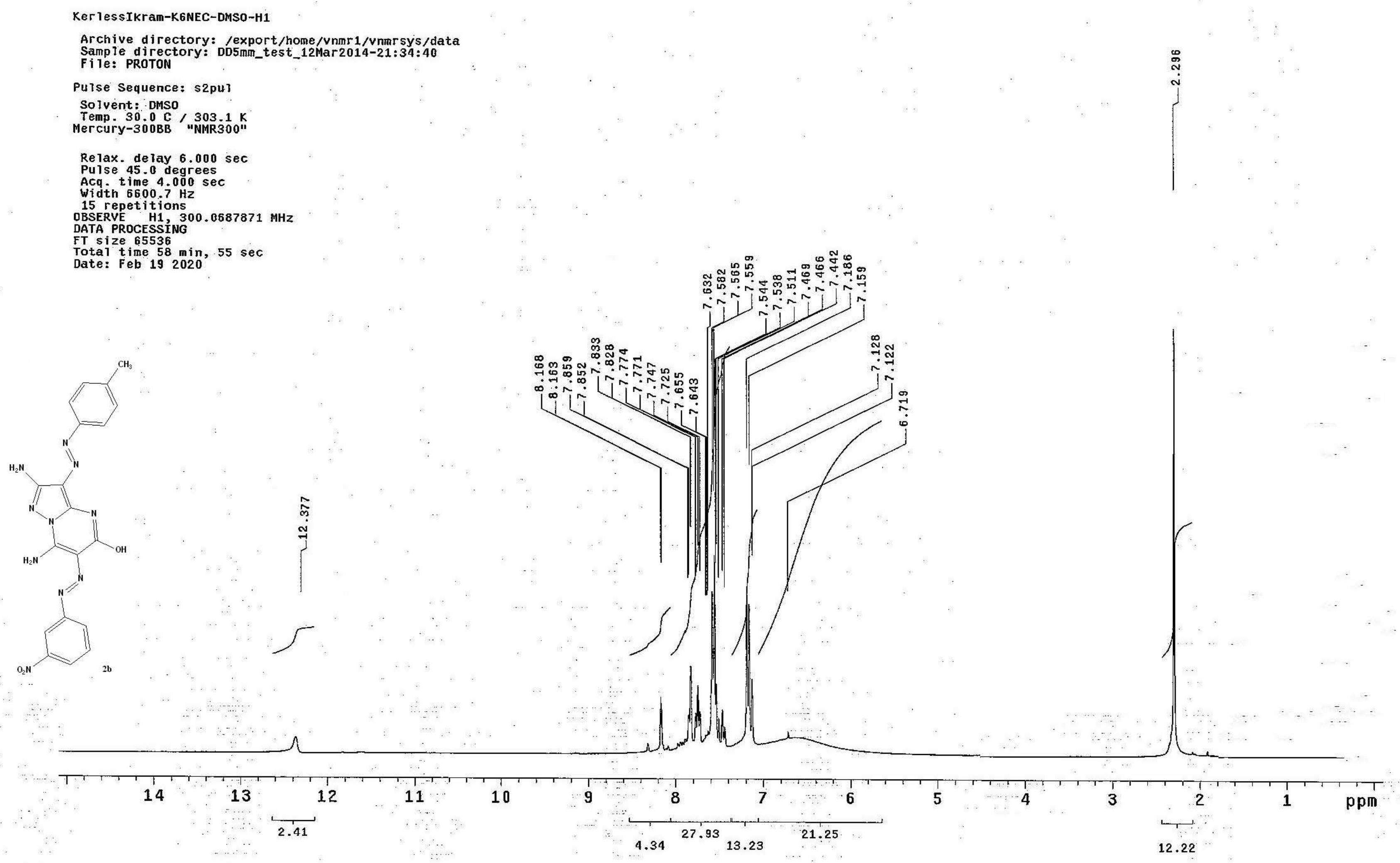

1H-NMR-D2O spectrum of compound 2b

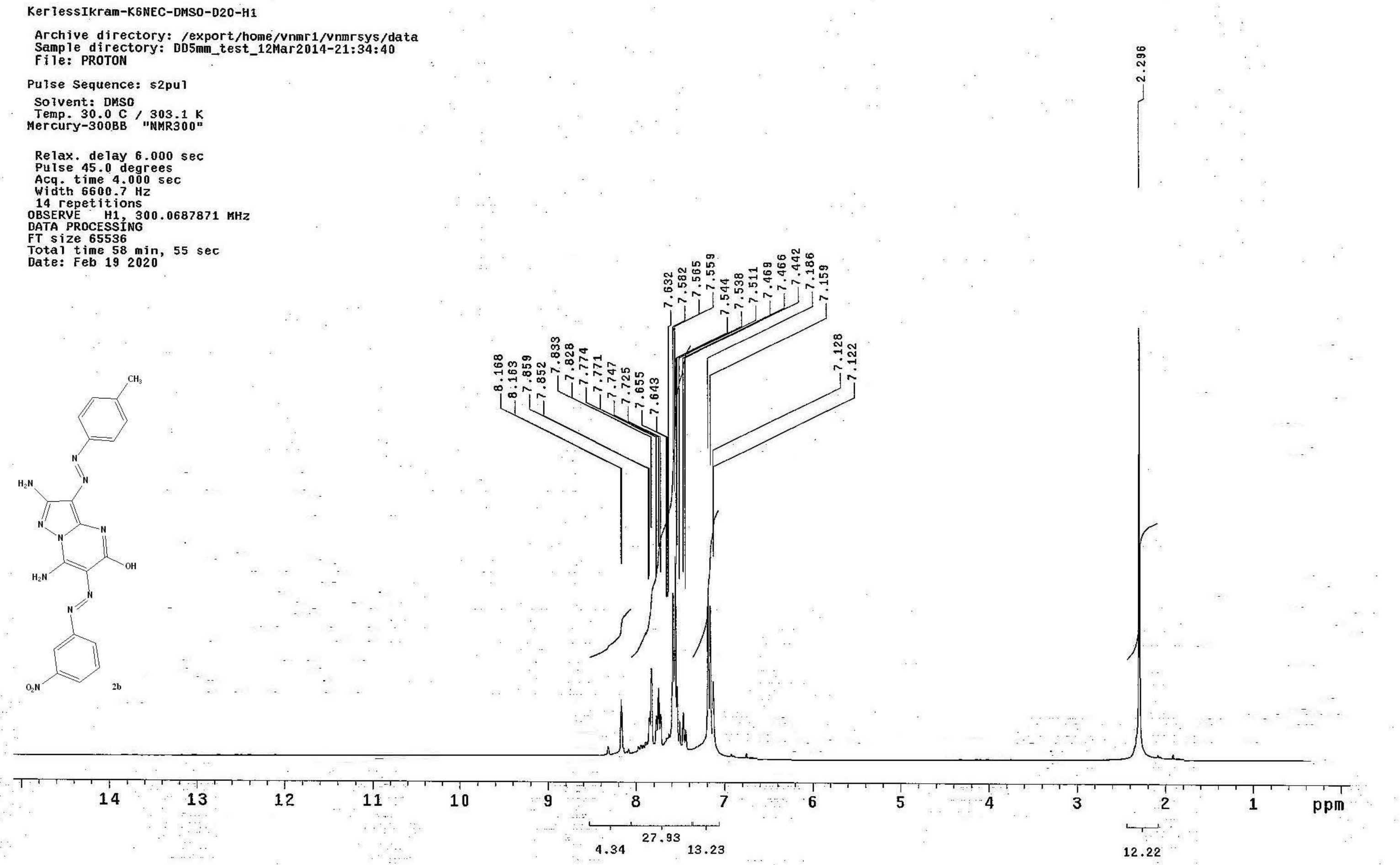

13C-NMR spectrum of compound 2b

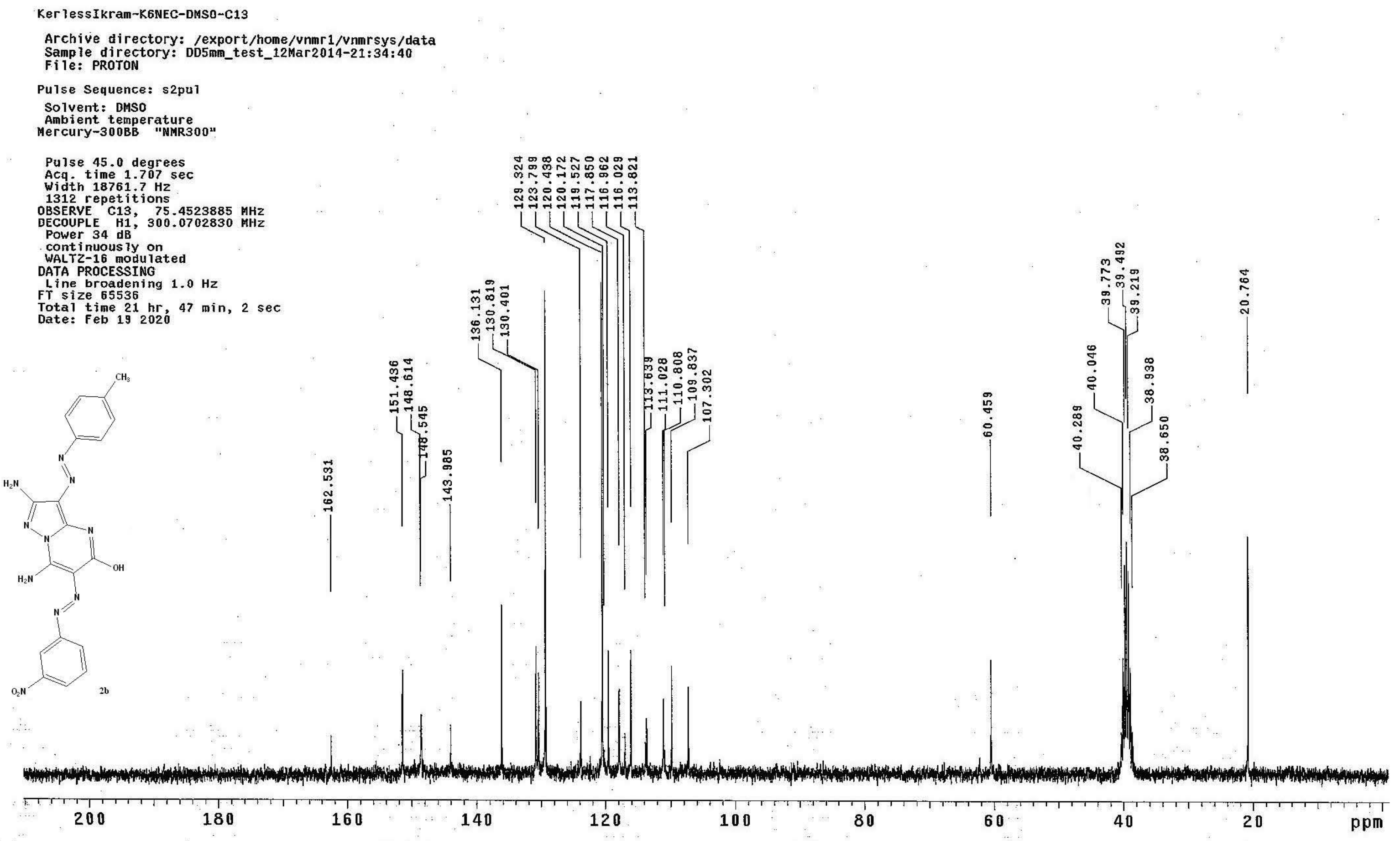

mass spectrum of compound 2b

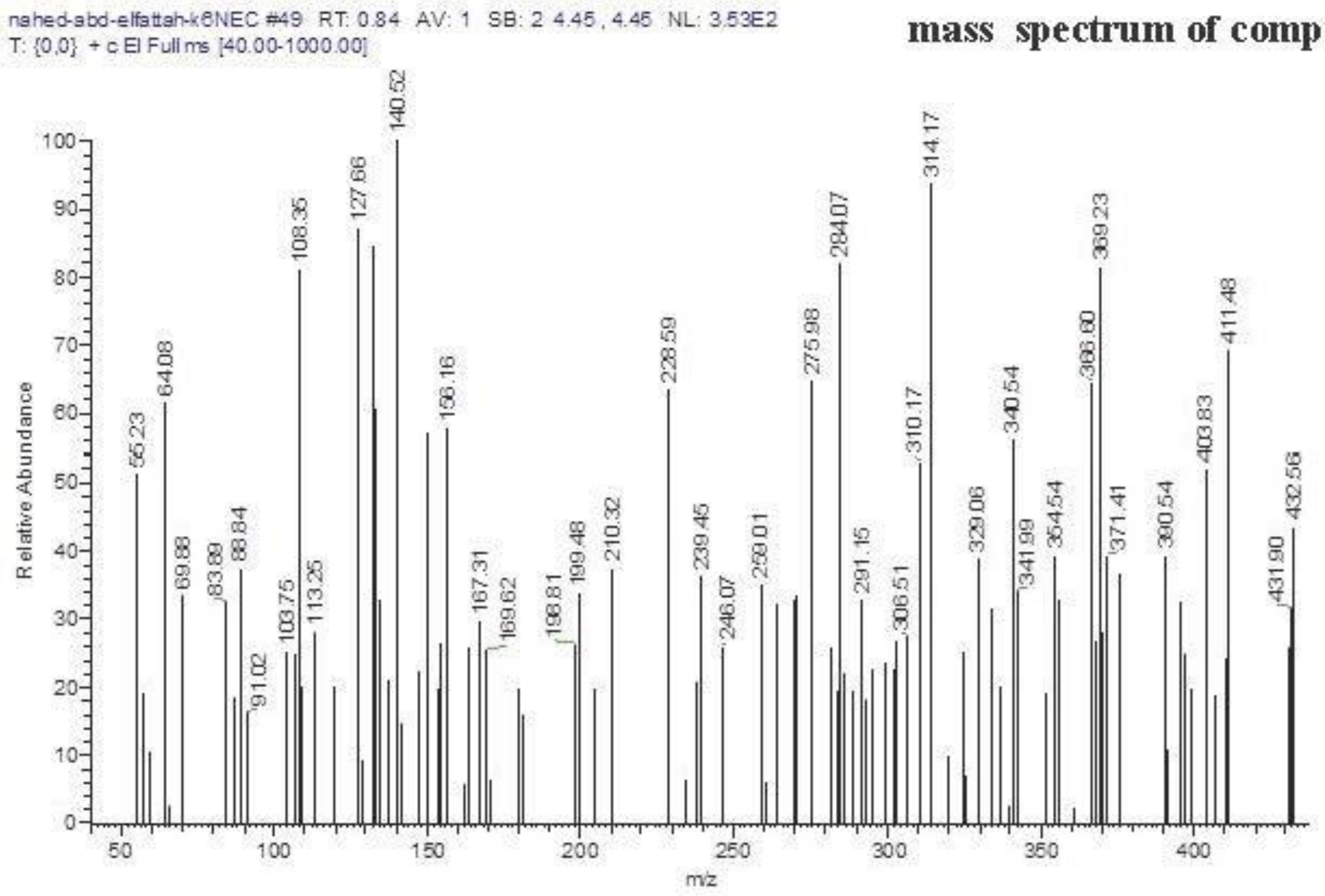

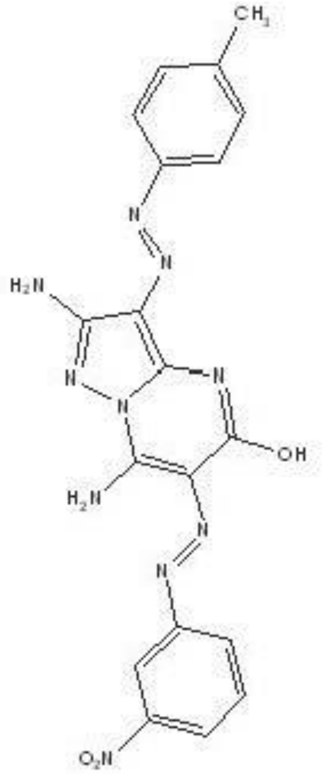

IR spectrum of compound 3a

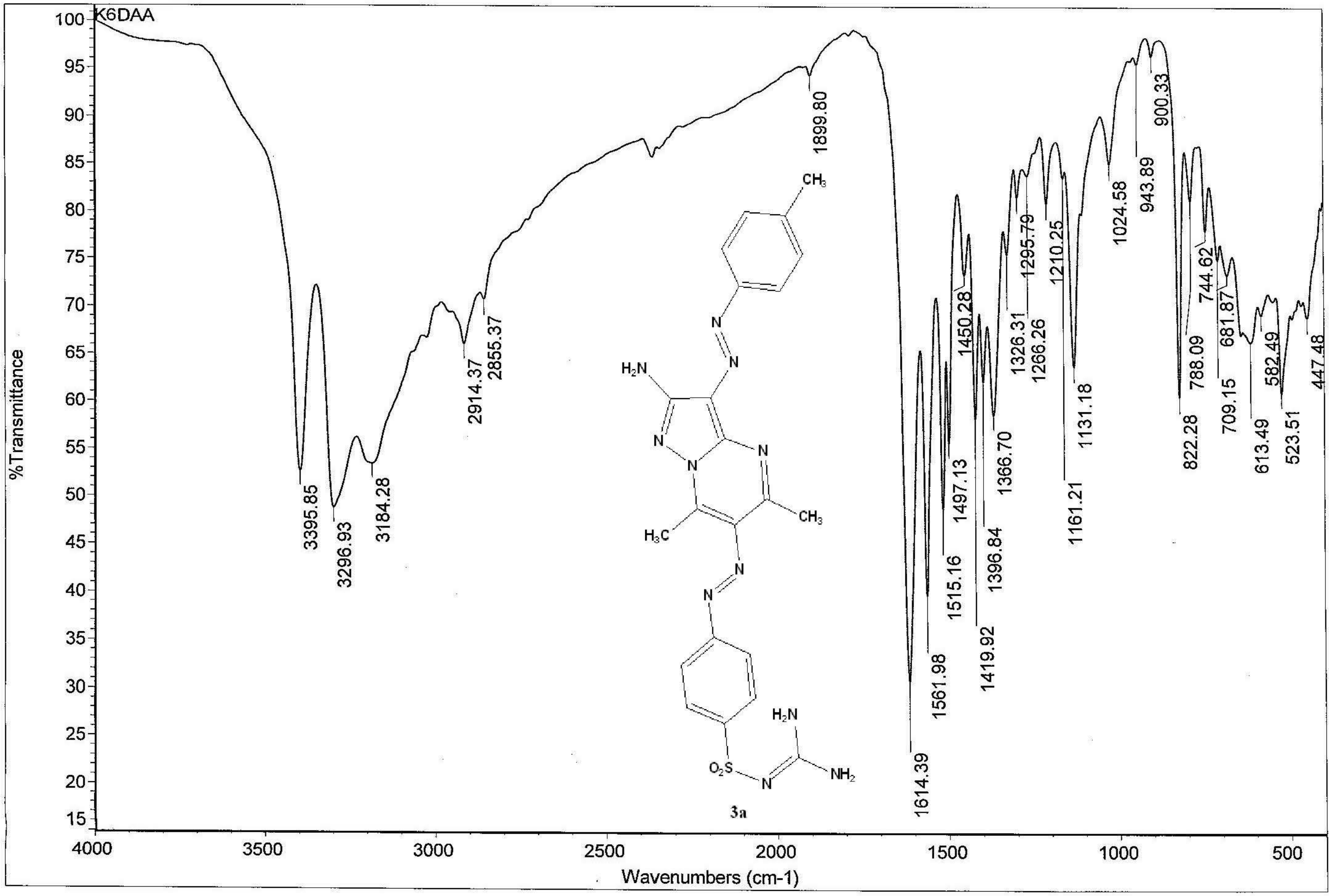

mass spectrum of compound 3a

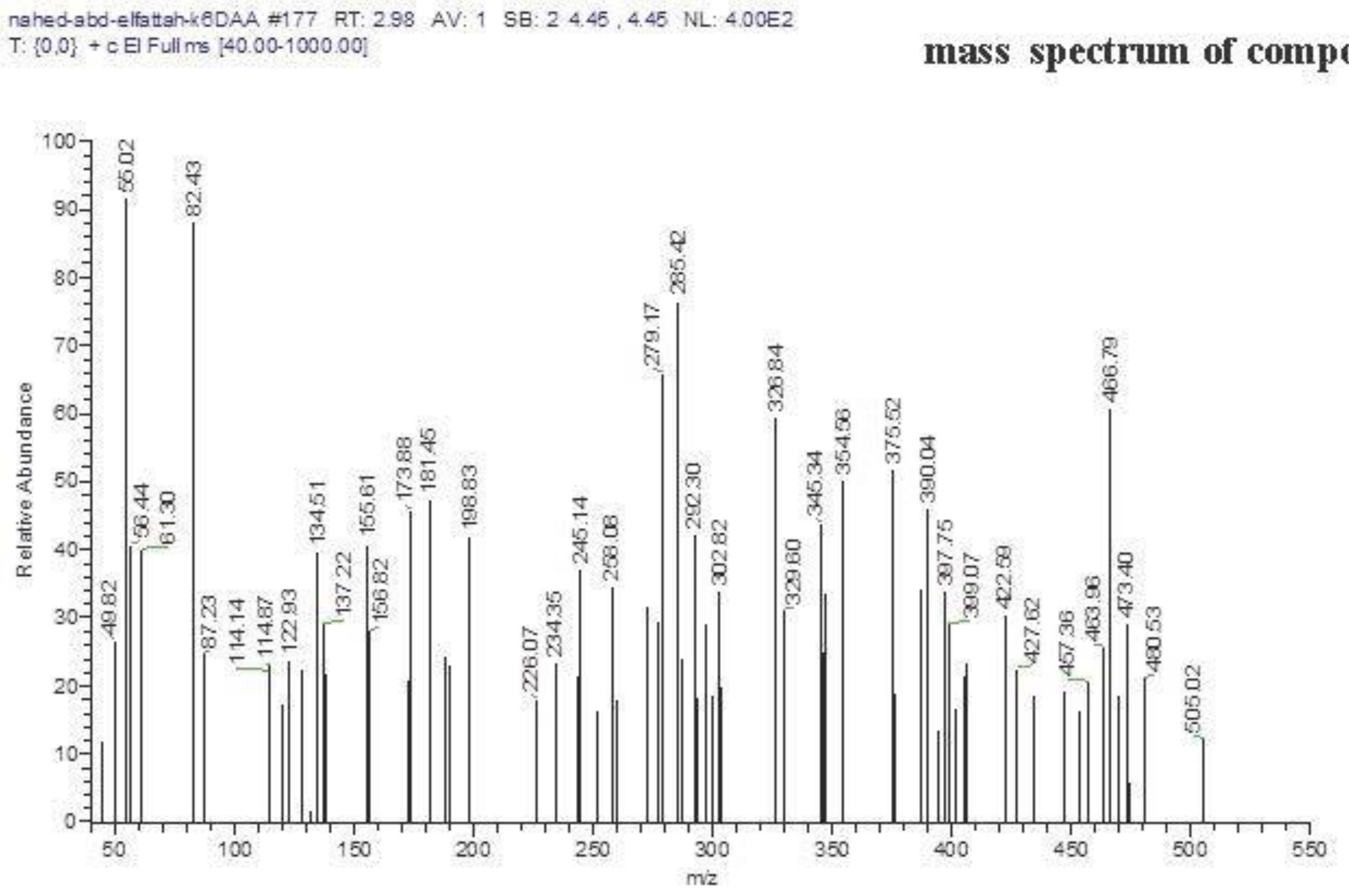

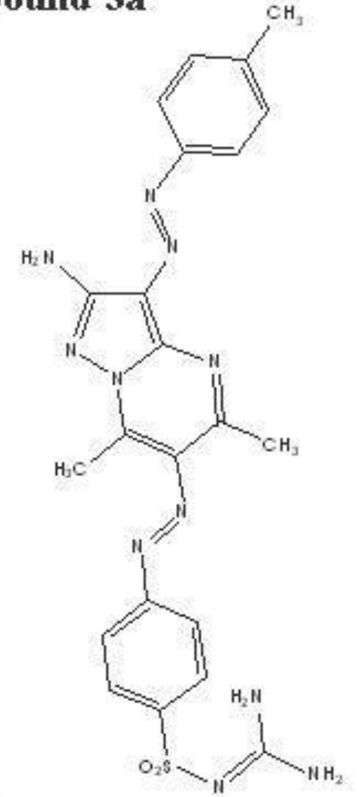

3a

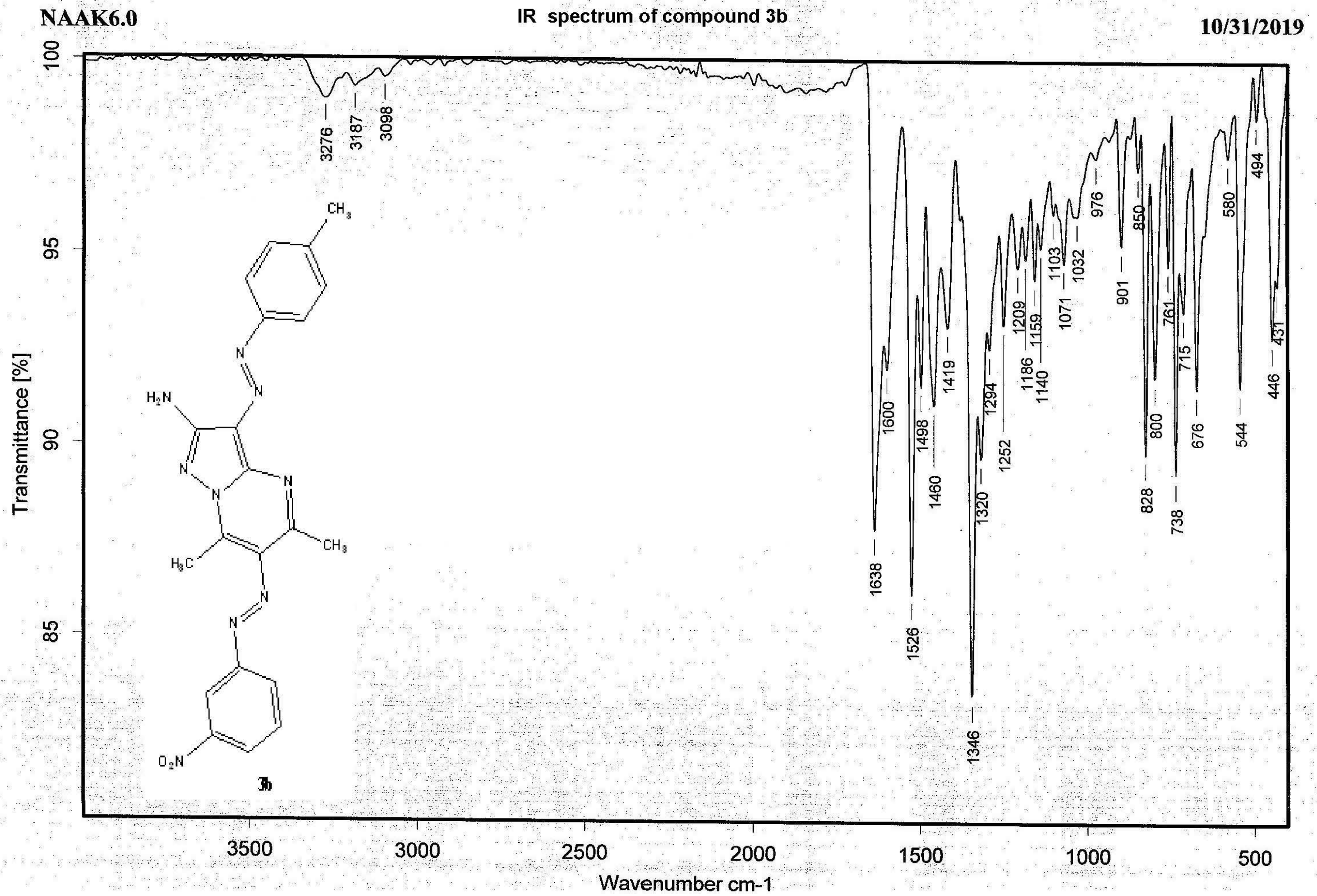
NAAK6.0
IR spectrum of compound 3b
10/31/2019
Transmittance [%]
100
95
90
85
Wavenumber cm-1
3500
3000
2500
2000
1500
1000
500
3276
3187
3098
1638
1600
1526
1498
1460
1419
1346
1320
1294
1252
1209
1186
1159
1140
1103
1071
1032
976
901
850
828
800
761
738
715
676
580
544
494
446
431
CH3
H2N
N
CH3
H3C
O2N

1H-NMR spectrum of compound 3b

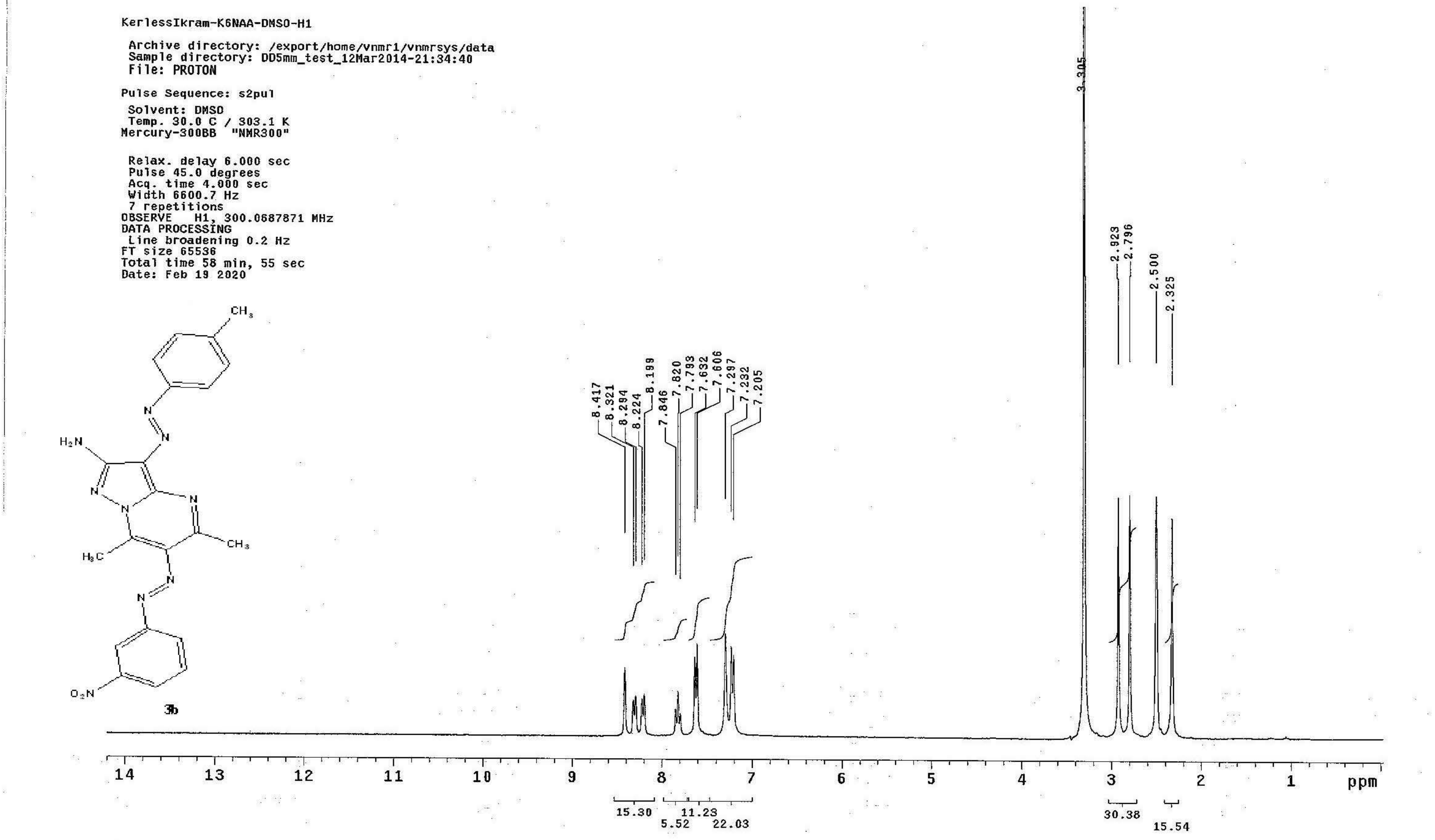

3b

1H-NMR-D2O spectrum of compound 3b

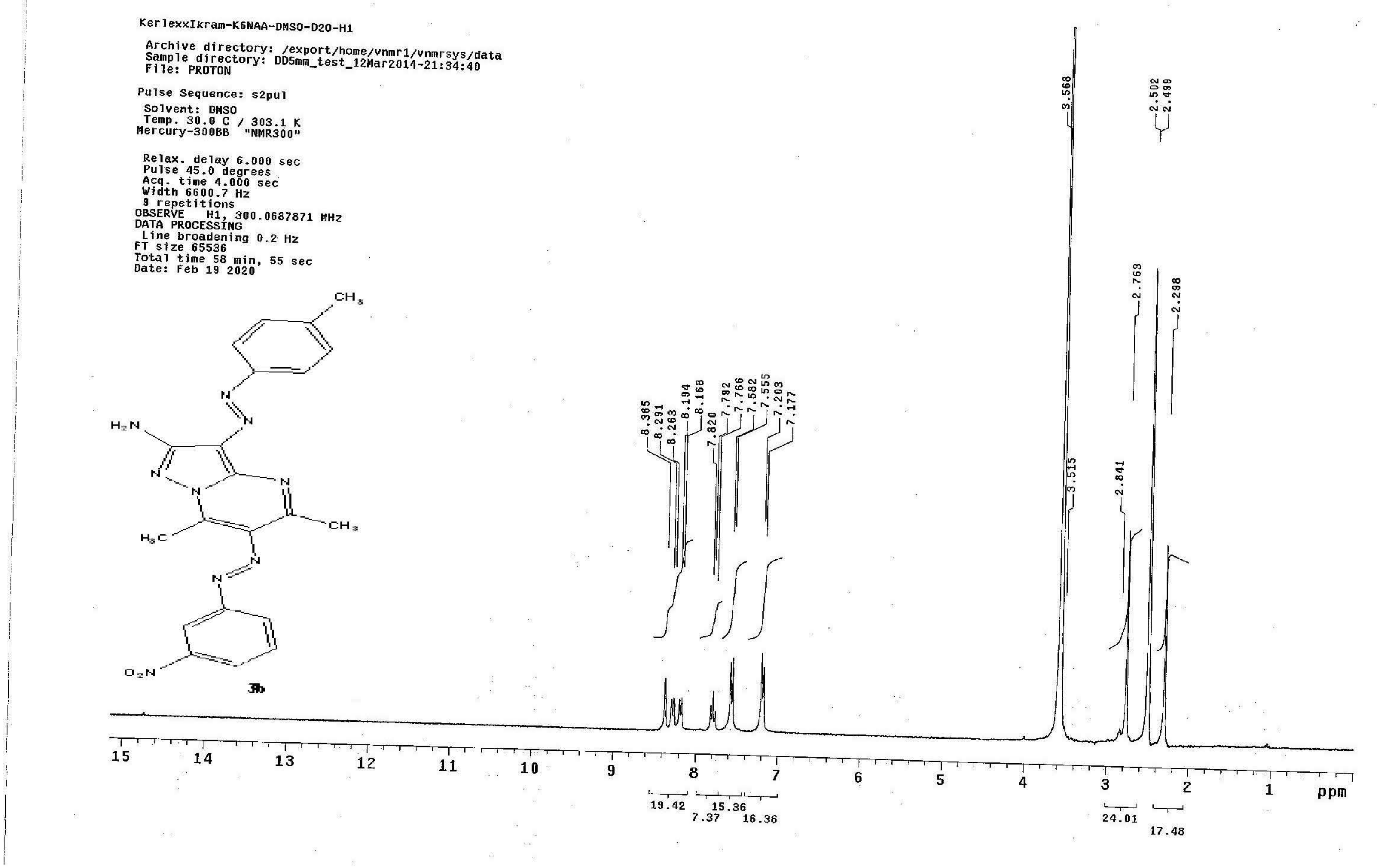

13C-NMR spectrum of compound 3b

```
KerlessIkram-K6NAA-DMSO-C13

 Archive directory: /export/home/vnmr1/vnmrsys/data
 Sample directory: DD5mm_test_12Mar2014-21:34:40
 File: PROTON

Pulse Sequence: s2pul
 Solvent: DMSO
 Ambient temperature
Mercury-300BB  "NMR300"

 Pulse 45.0 degrees
 Acq. time 1.707 sec
 Width 18761.7 Hz
 1312 repetitions
OBSERVE  C13,  75.4523885 MHz
DECOUPLE  H1, 300.0702830 MHz
 Power 34 dB
 continuously on
 WALTZ-16 modulated
DATA PROCESSING
 Line broadening 1.0 Hz
FT size 65536
Total time 21 hr, 47 min, 2 sec
Date: Feb 19 2020
```

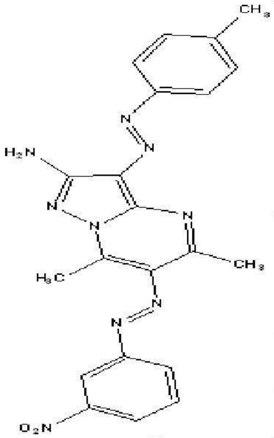

3b

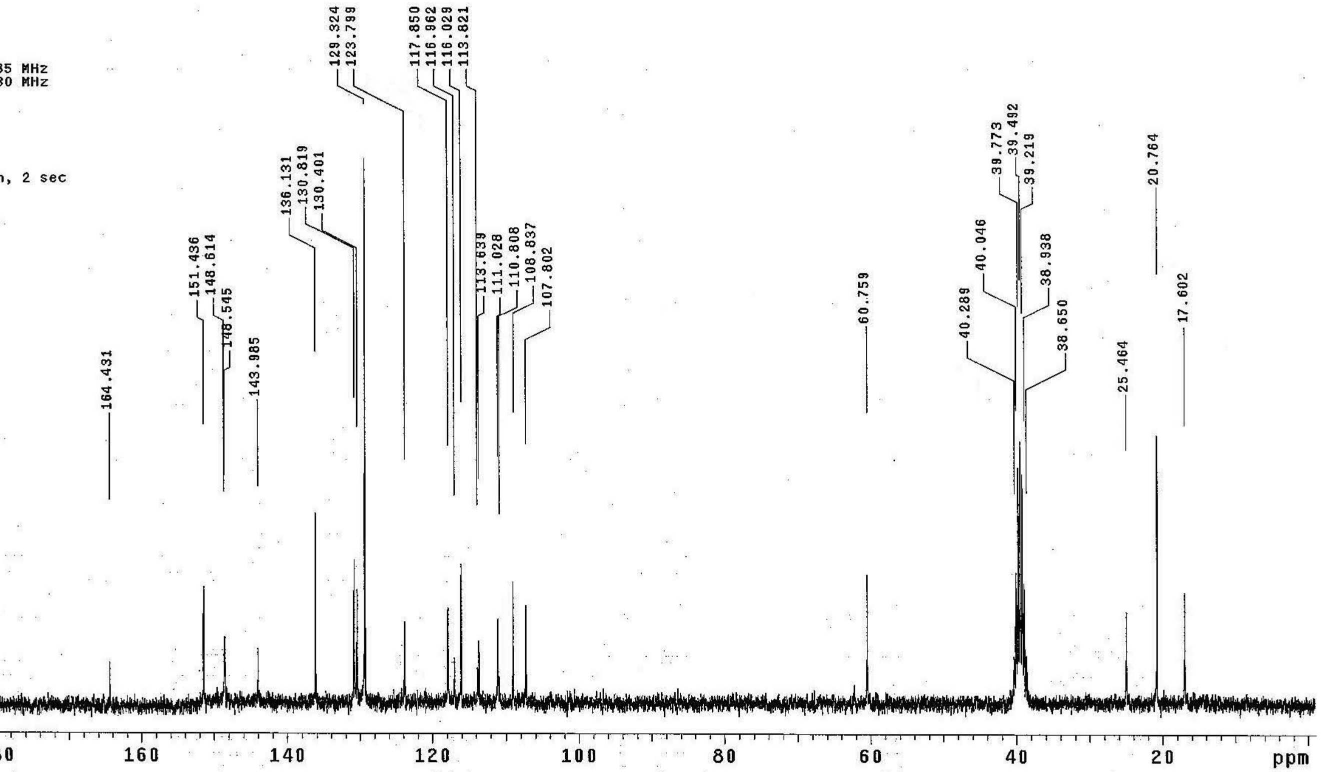

mass spectrum of compound 3b

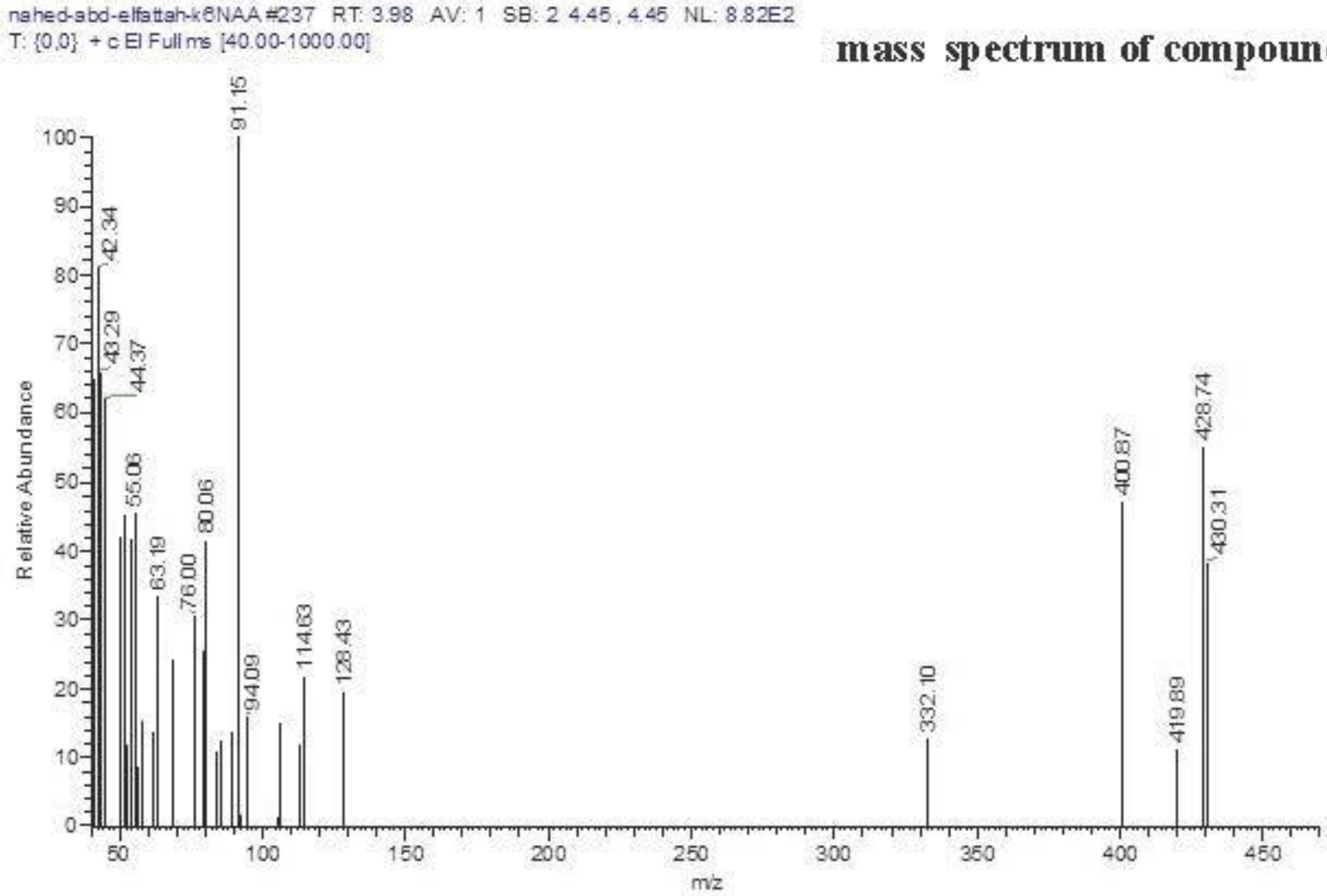

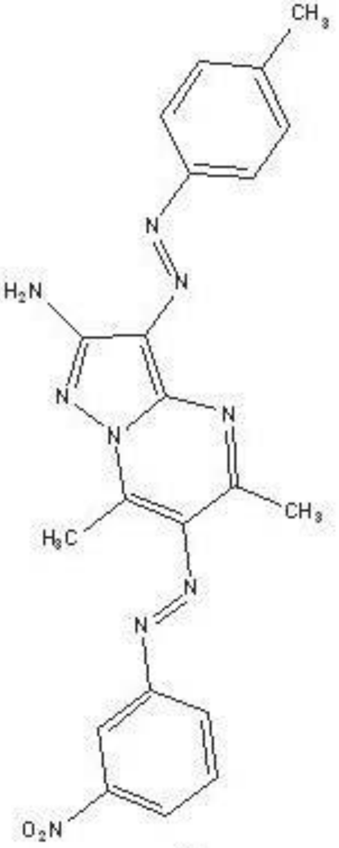

3b

IR spectrum of compound 4a

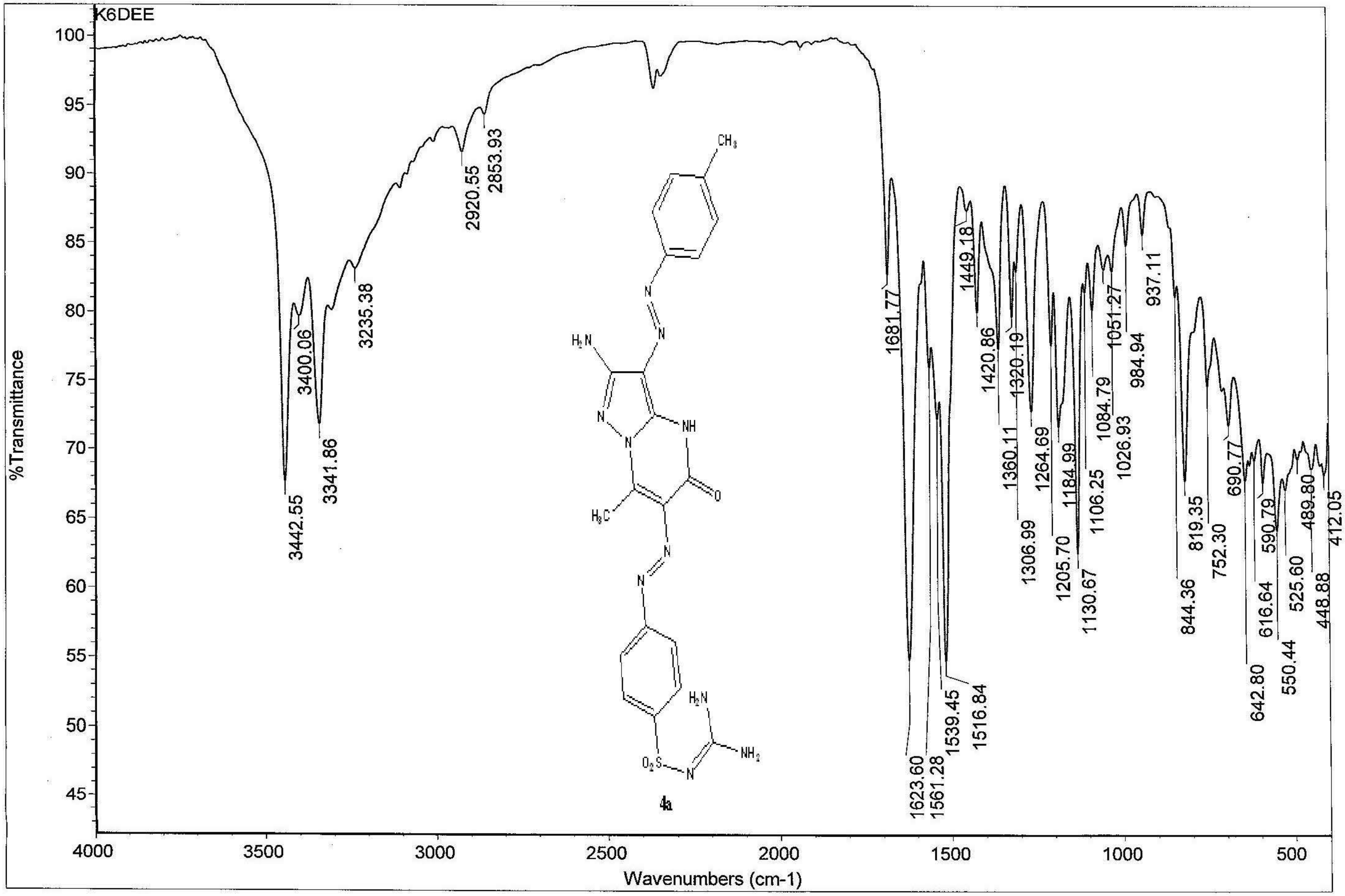

mass spectrum of compound 4a

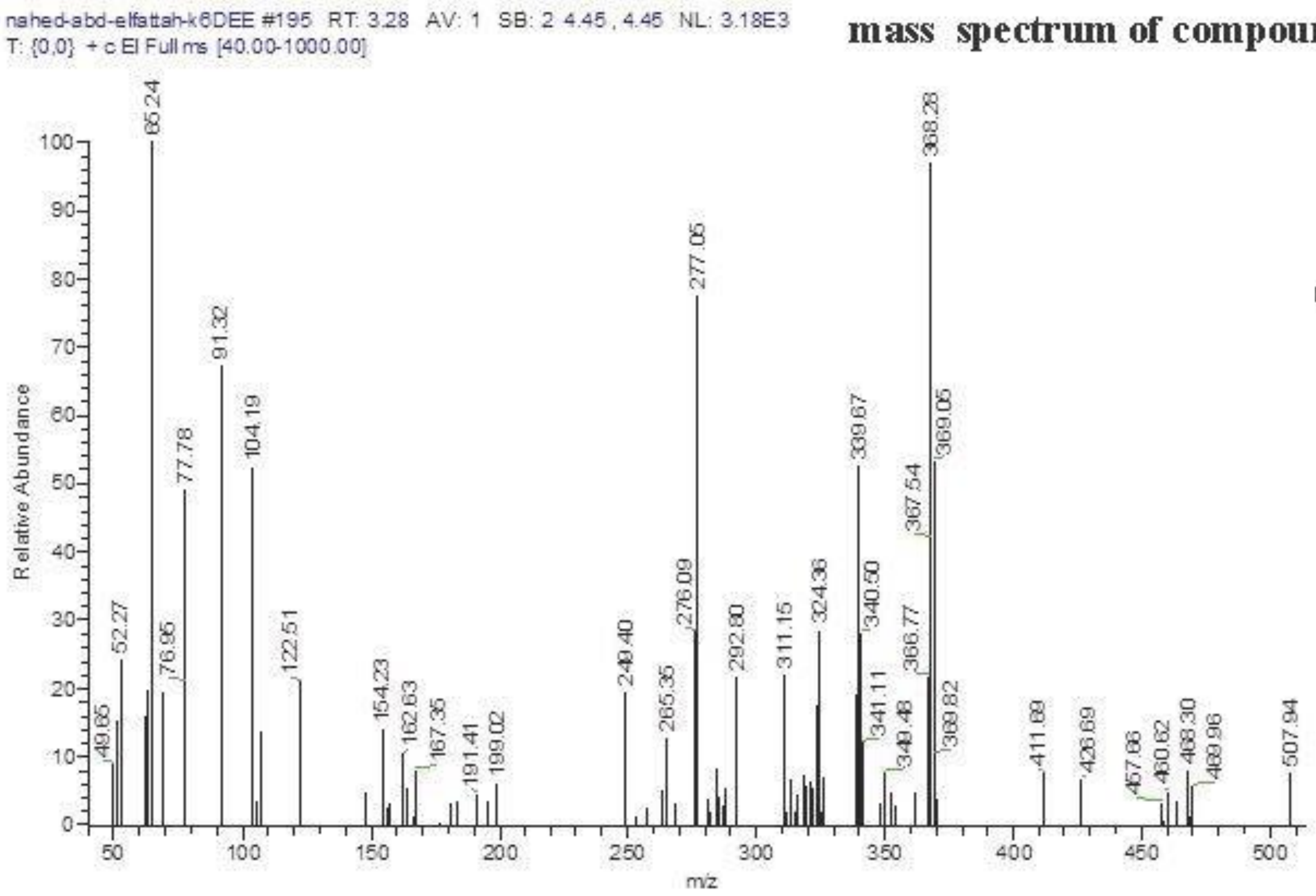

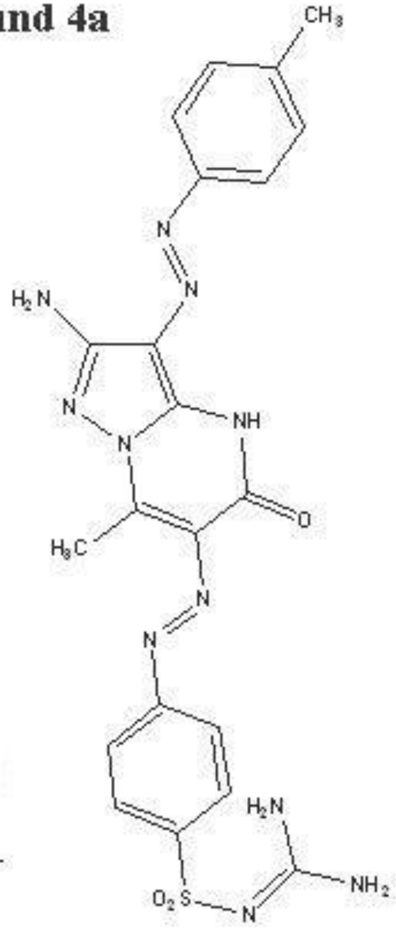

4a

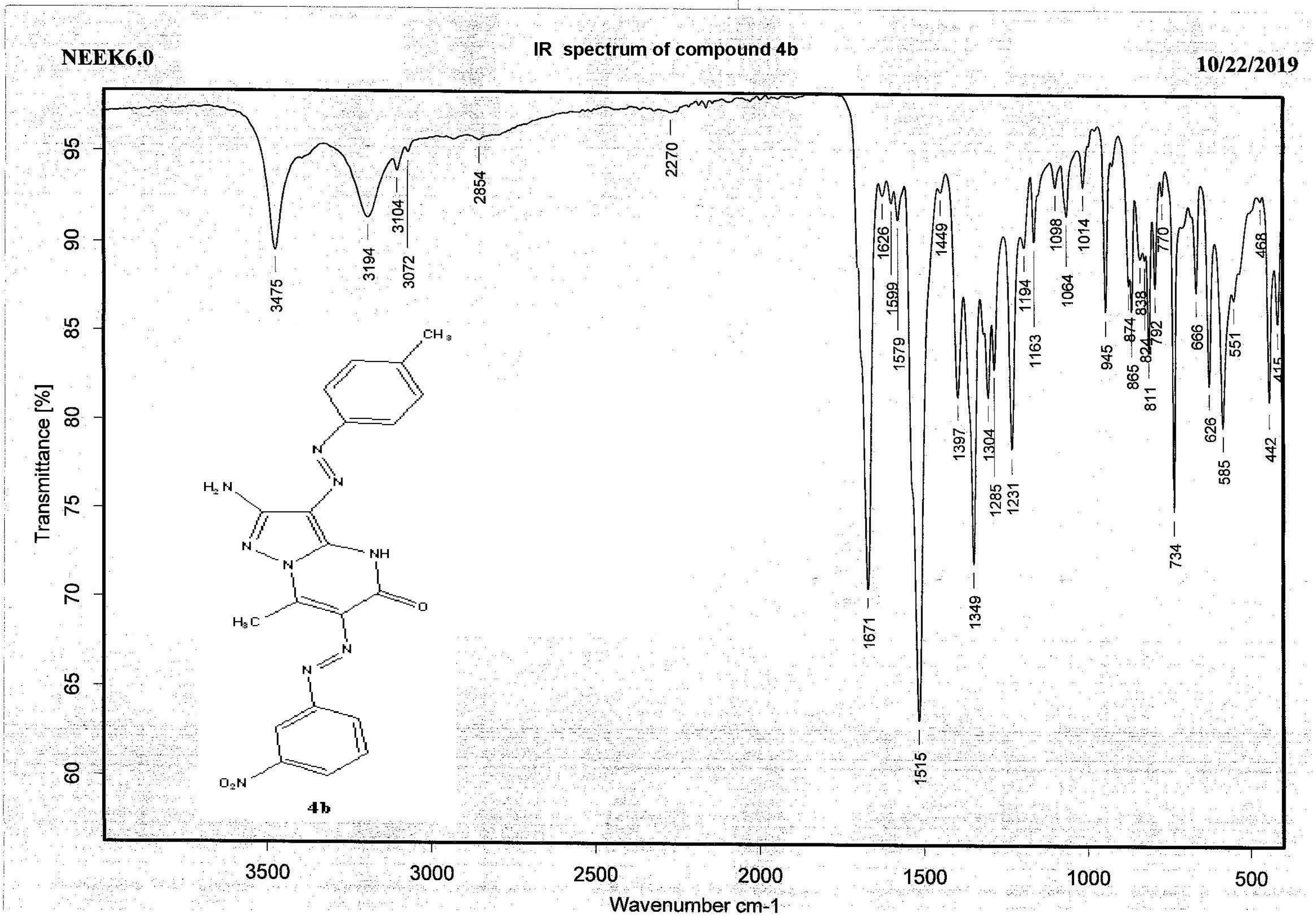
NEEK6.0
IR spectrum of compound 4b
10/22/2019
Transmittance [%]
Wavenumber cm-1
3475
3194
3104
3072
2854
2270
1671
1626
1599
1579
1515
1449
1397
1349
1304
1285
1231
1194
1163
1098
1064
1014
945
874
865
838
824
811
792
770
734
666
626
585
551
468
442
415
4b

1H-NMR spectrum of compound 4b

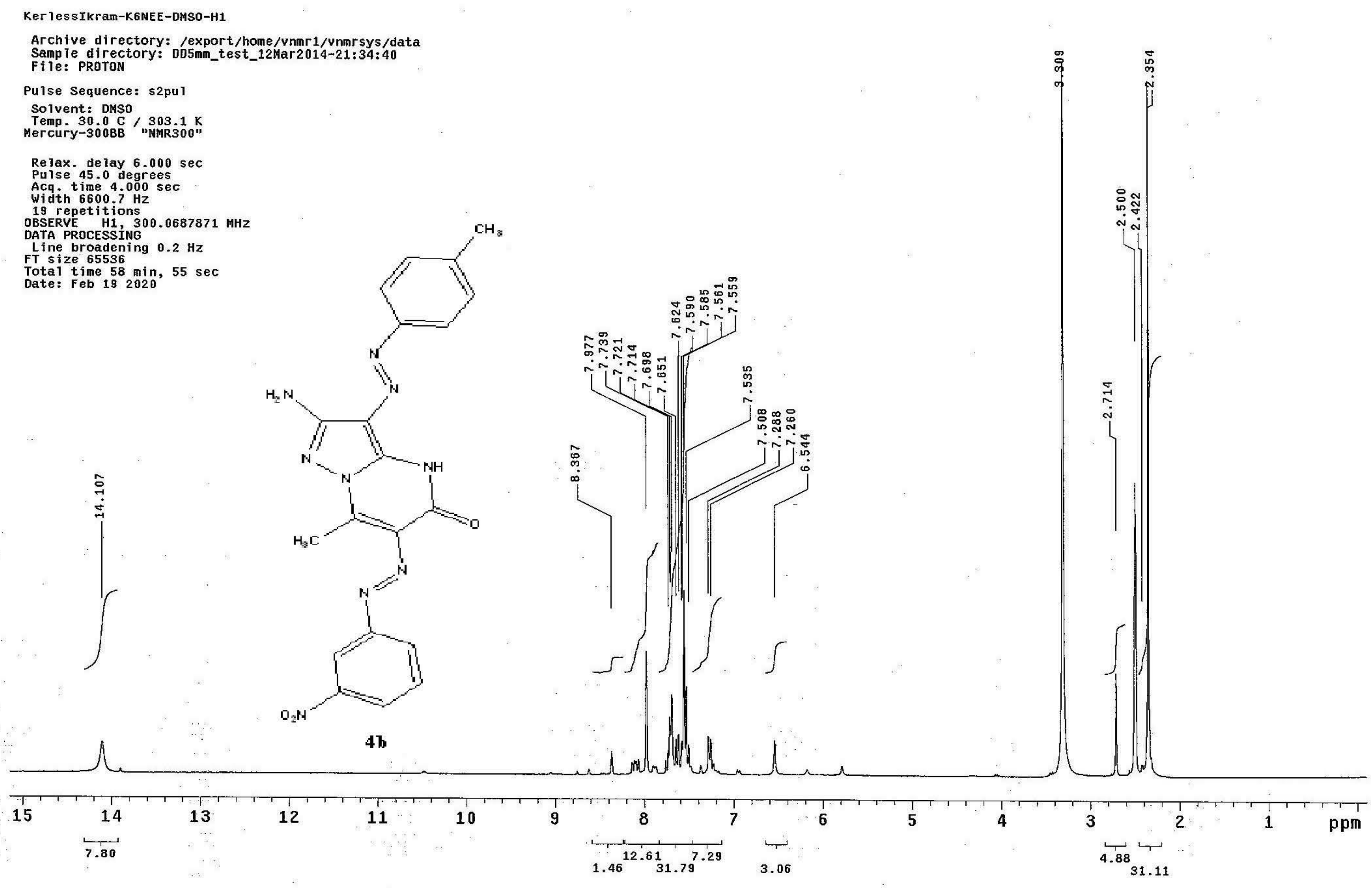

1H-NMR-D2O spectrum of compound 4b

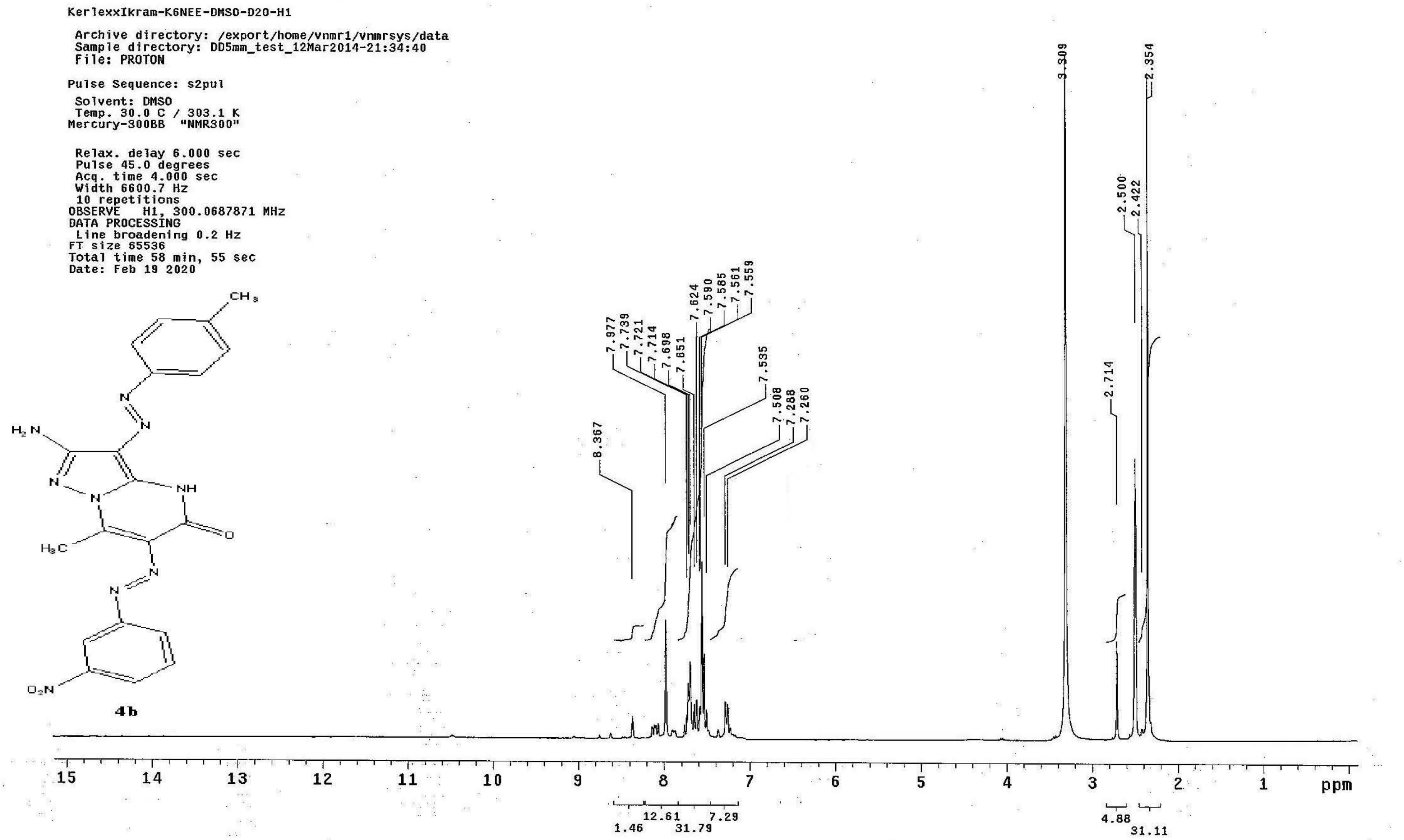

13C-NMR spectrum of compound 4b

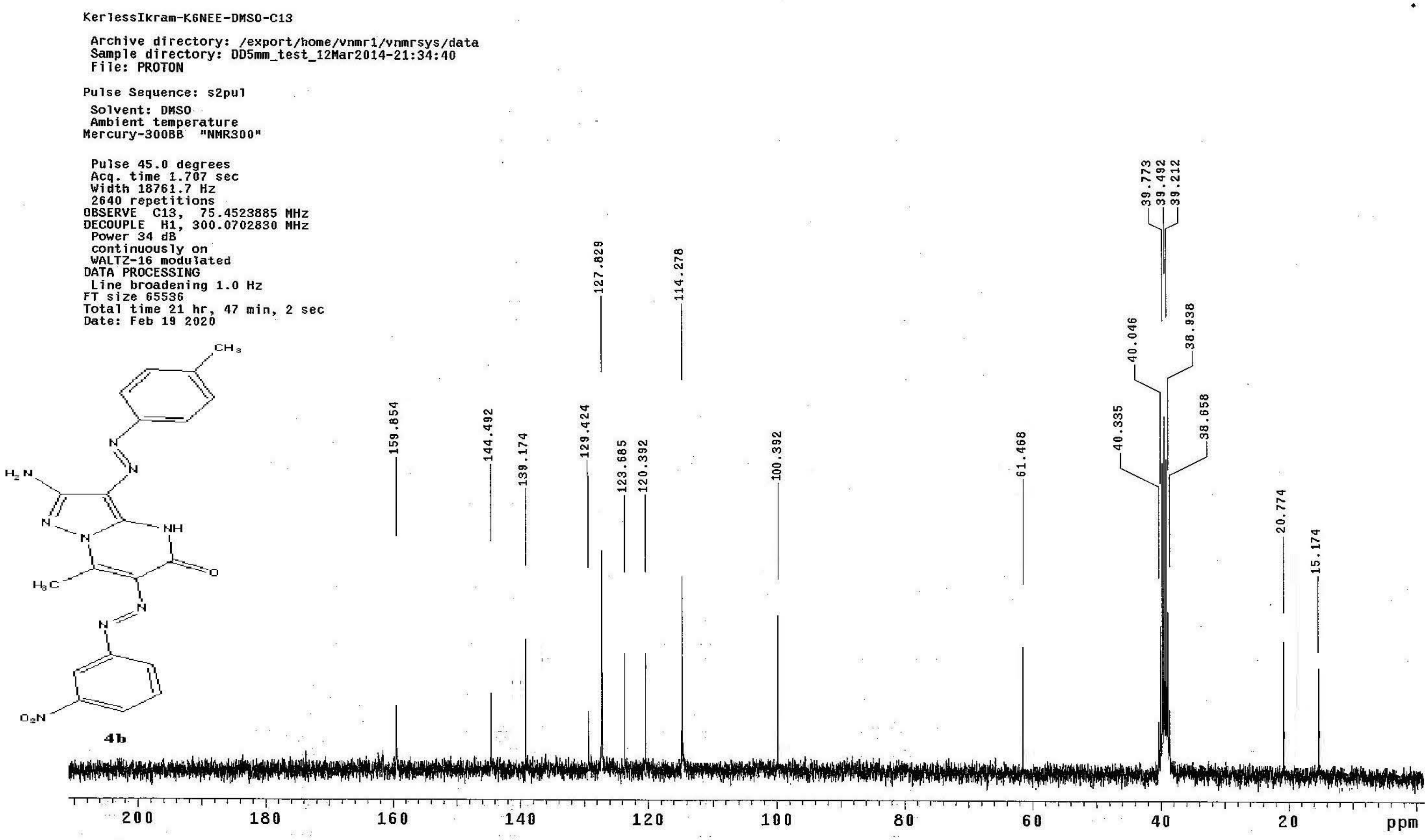

mass spectrum of compound 4b

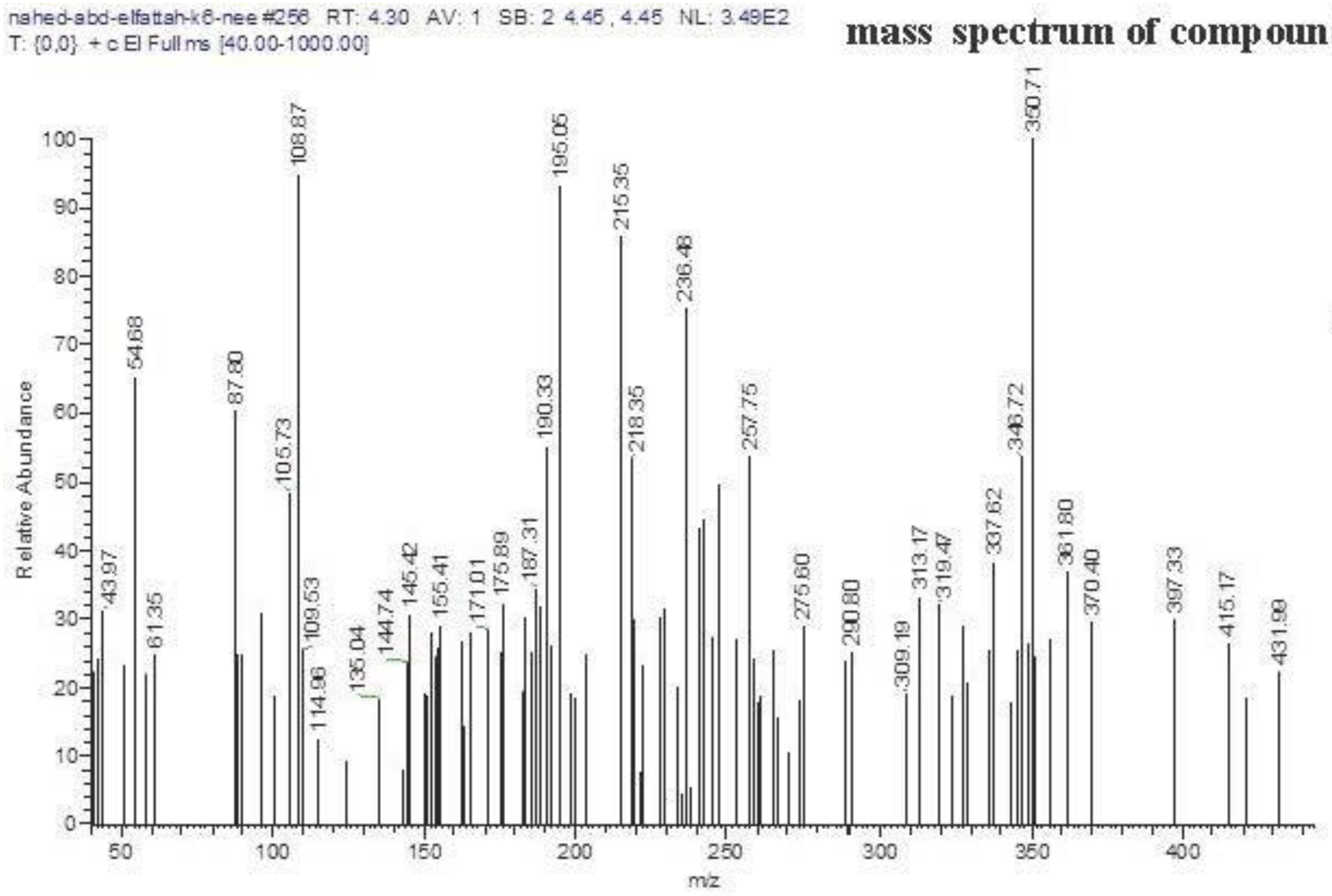

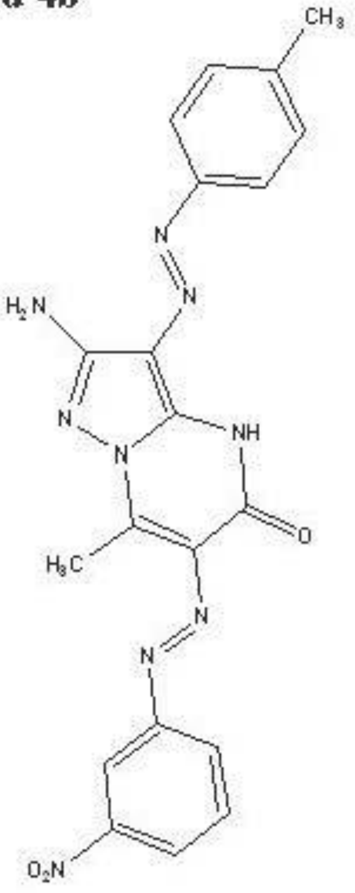

4b

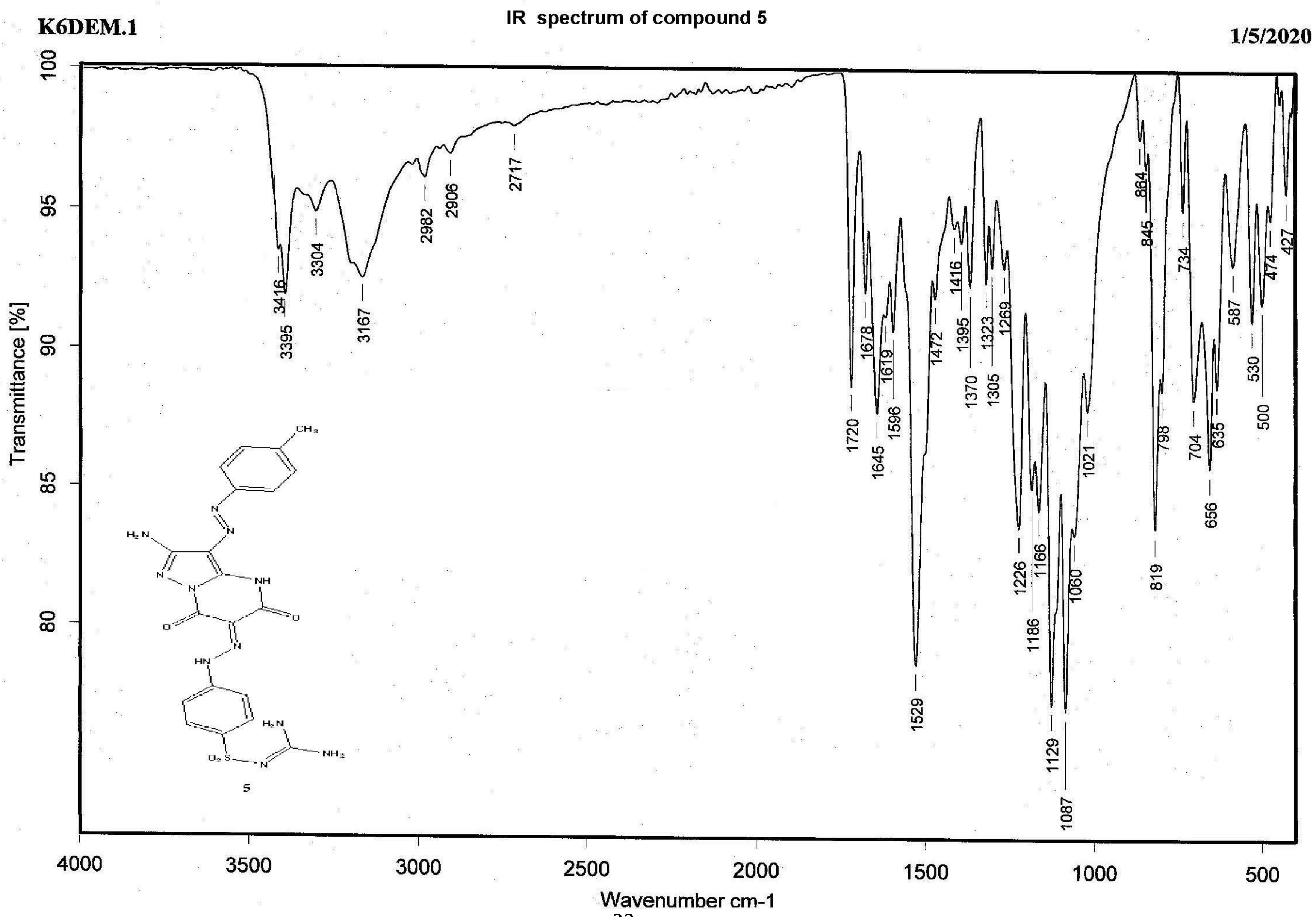
K6DEM.1
IR spectrum of compound 5
1/5/2020
Transmittance [%]
100
95
90
85
80
Wavenumber cm-1
4000
3500
3000
2500
2000
1500
1000
500
3416
3395
3304
3167
2982
2906
2717
1720
1678
1645
1619
1596
1529
1472
1416
1395
1370
1323
1305
1269
1226
1186
1166
1129
1087
1060
1021
864
845
819
798
734
704
656
635
587
530
500
474
427
CH3
H2N
N
NH
O
HN
NH2
O2S
5

1H-NMR spectrum of compound 5

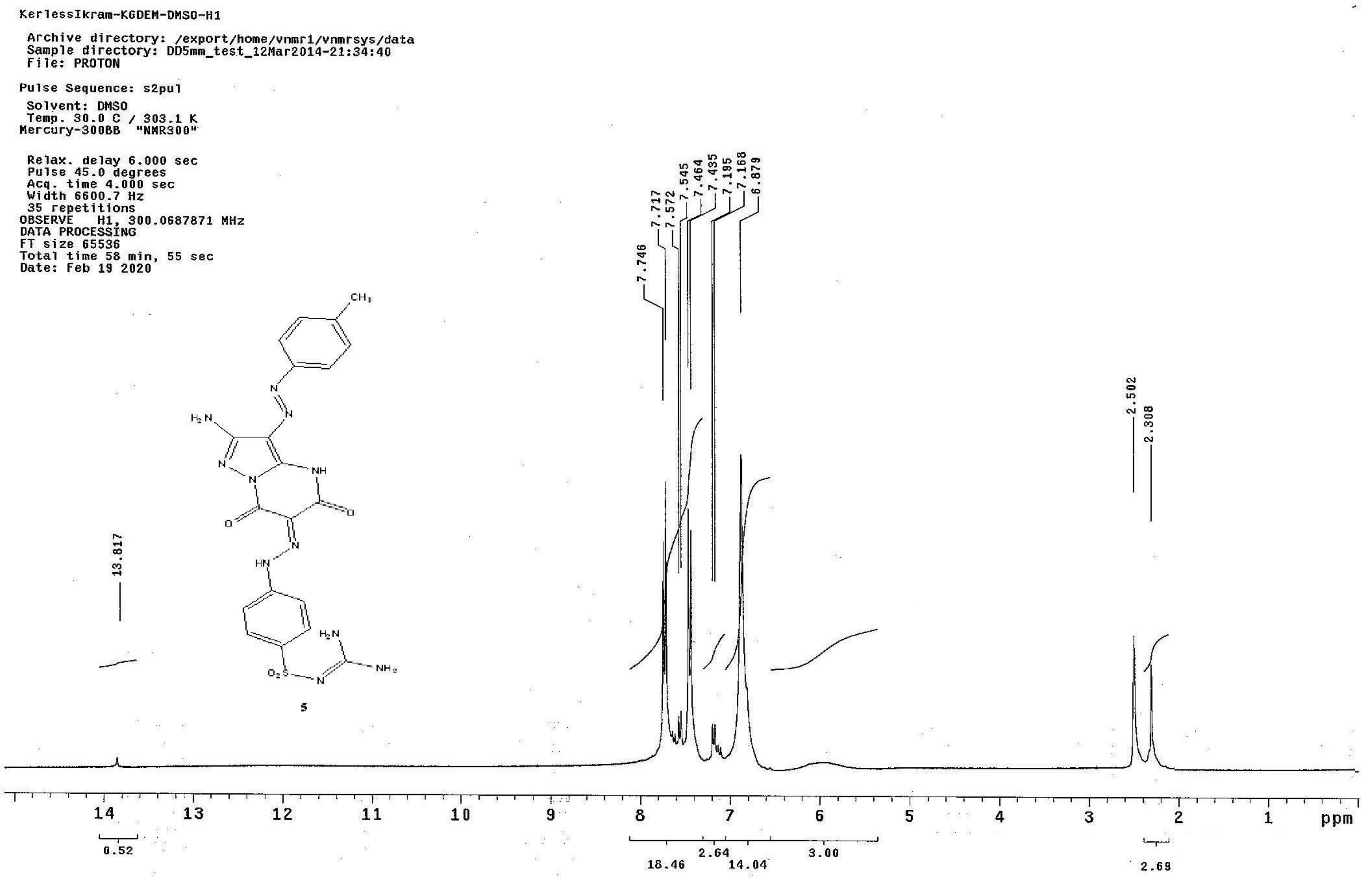

1H-NMR-D2O spectrum of compound 5

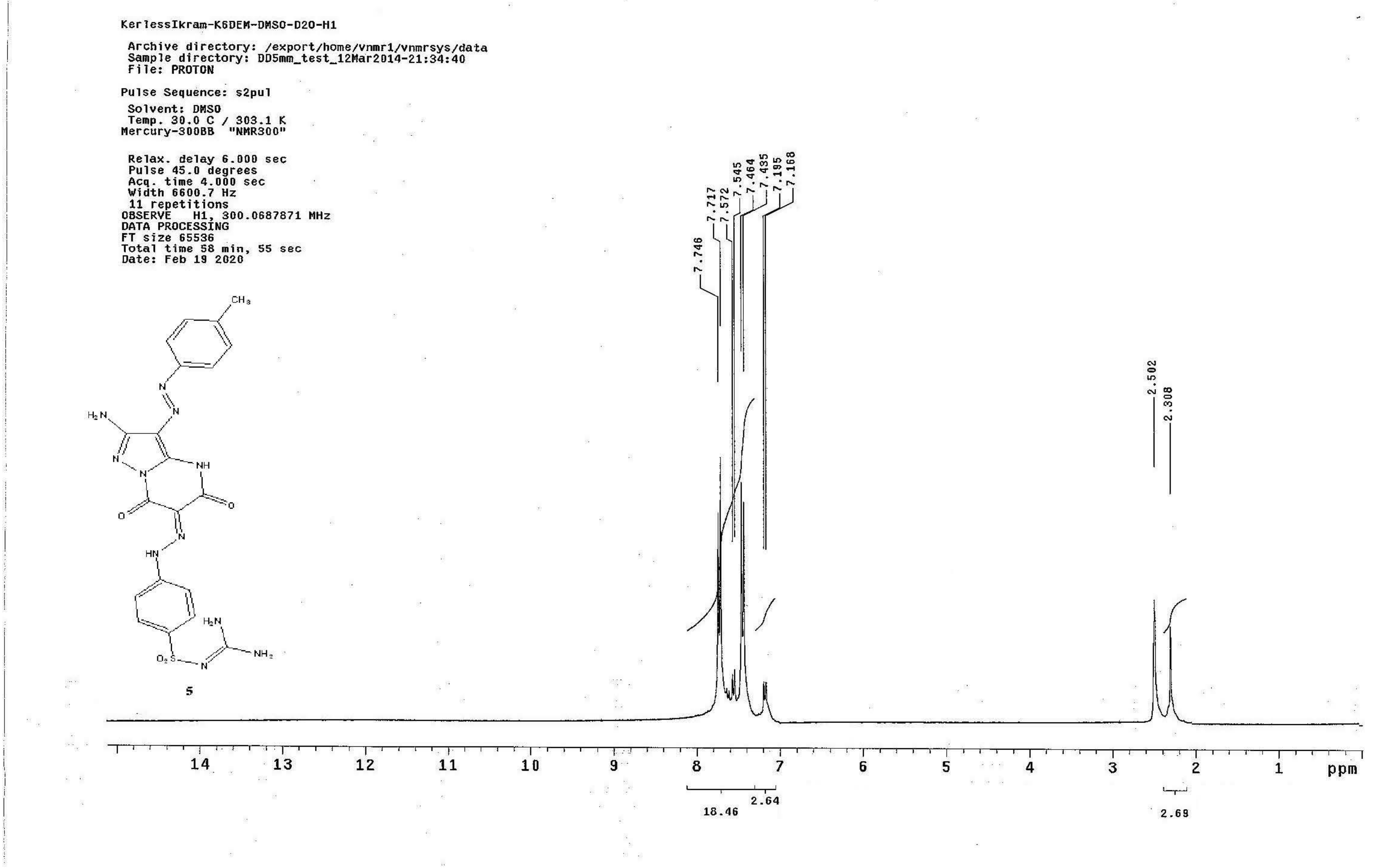

13C-NMR spectrum of compound 5

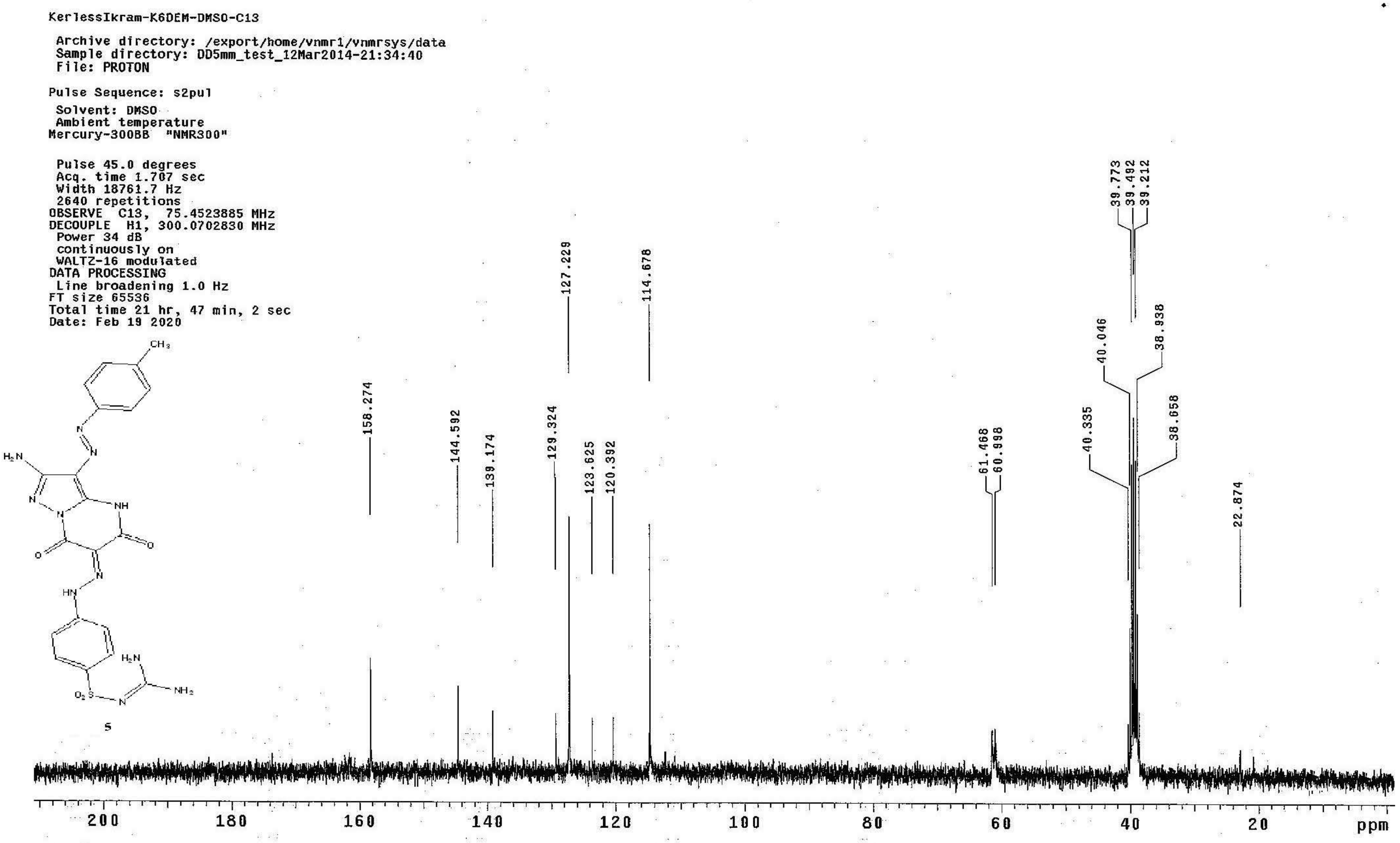

mass spectrum of compound 5

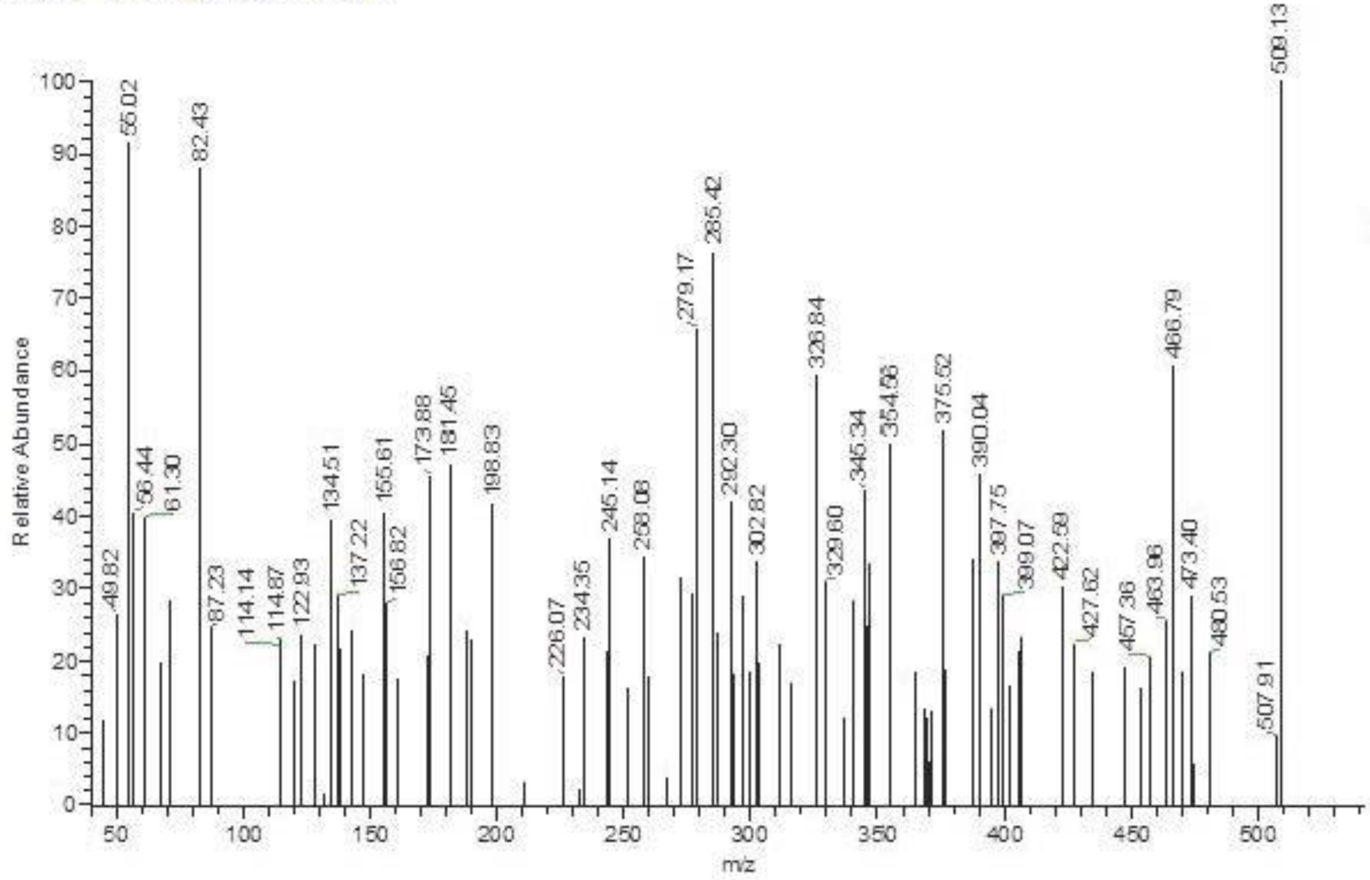

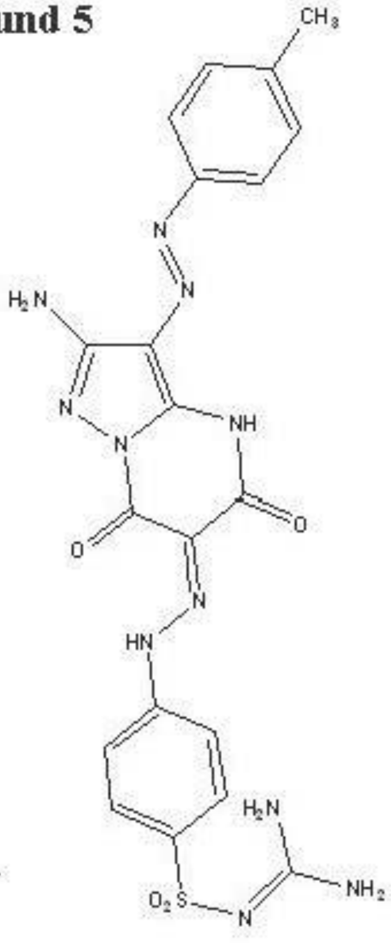

5

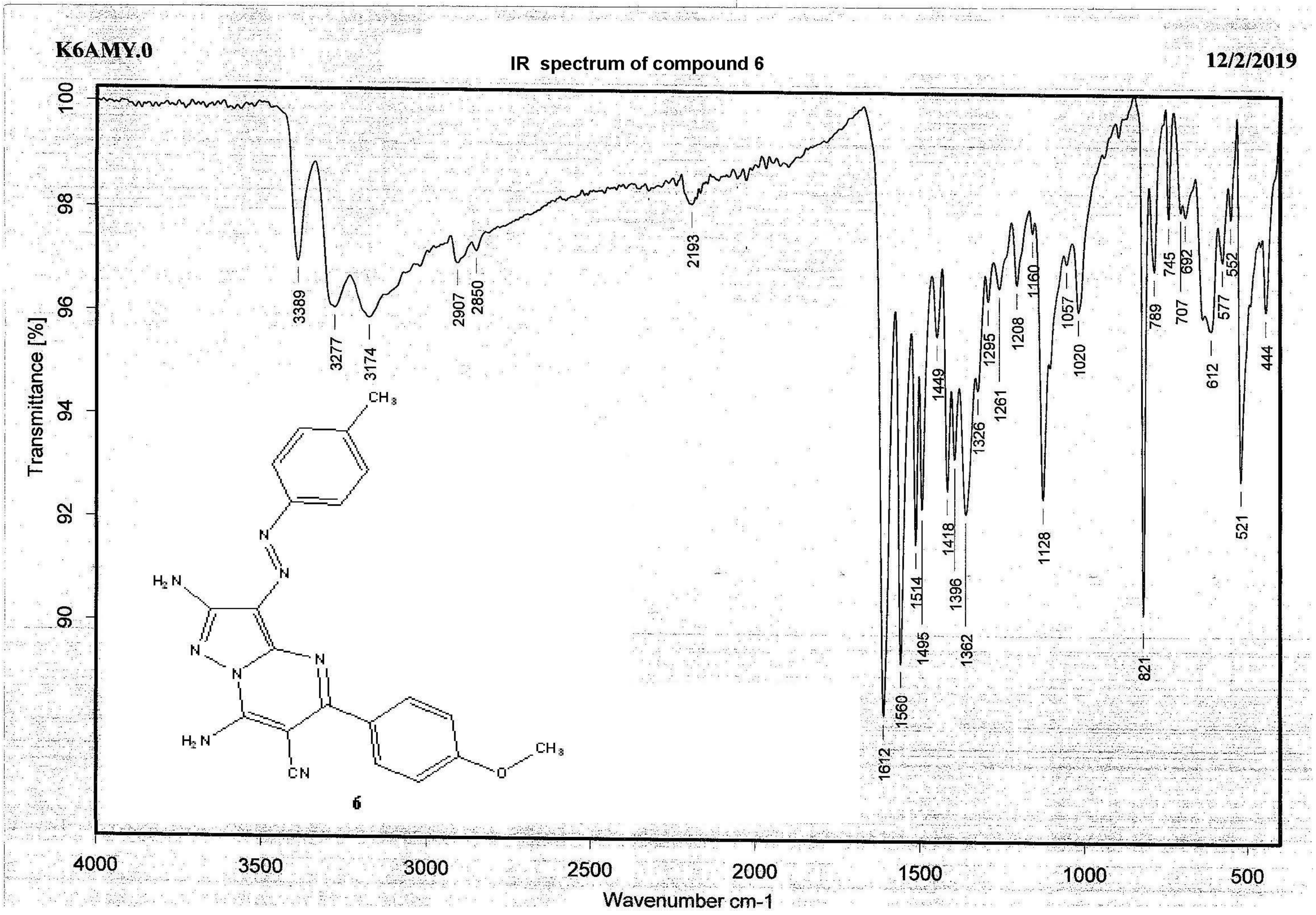
K6AMY.0
IR spectrum of compound 6
12/2/2019
Transmittance [%]
Wavenumber cm-1
100
98
96
94
92
90
4000
3500
3000
2500
2000
1500
1000
500
3389
3277
3174
2907
2850
2193
1612
1560
1514
1495
1449
1418
1396
1362
1326
1295
1261
1208
1160
1128
1057
1020
821
789
745
707
692
612
577
552
521
444
CH3
H2N
N
CN
O
6

1H-NMR spectrum of compound 6

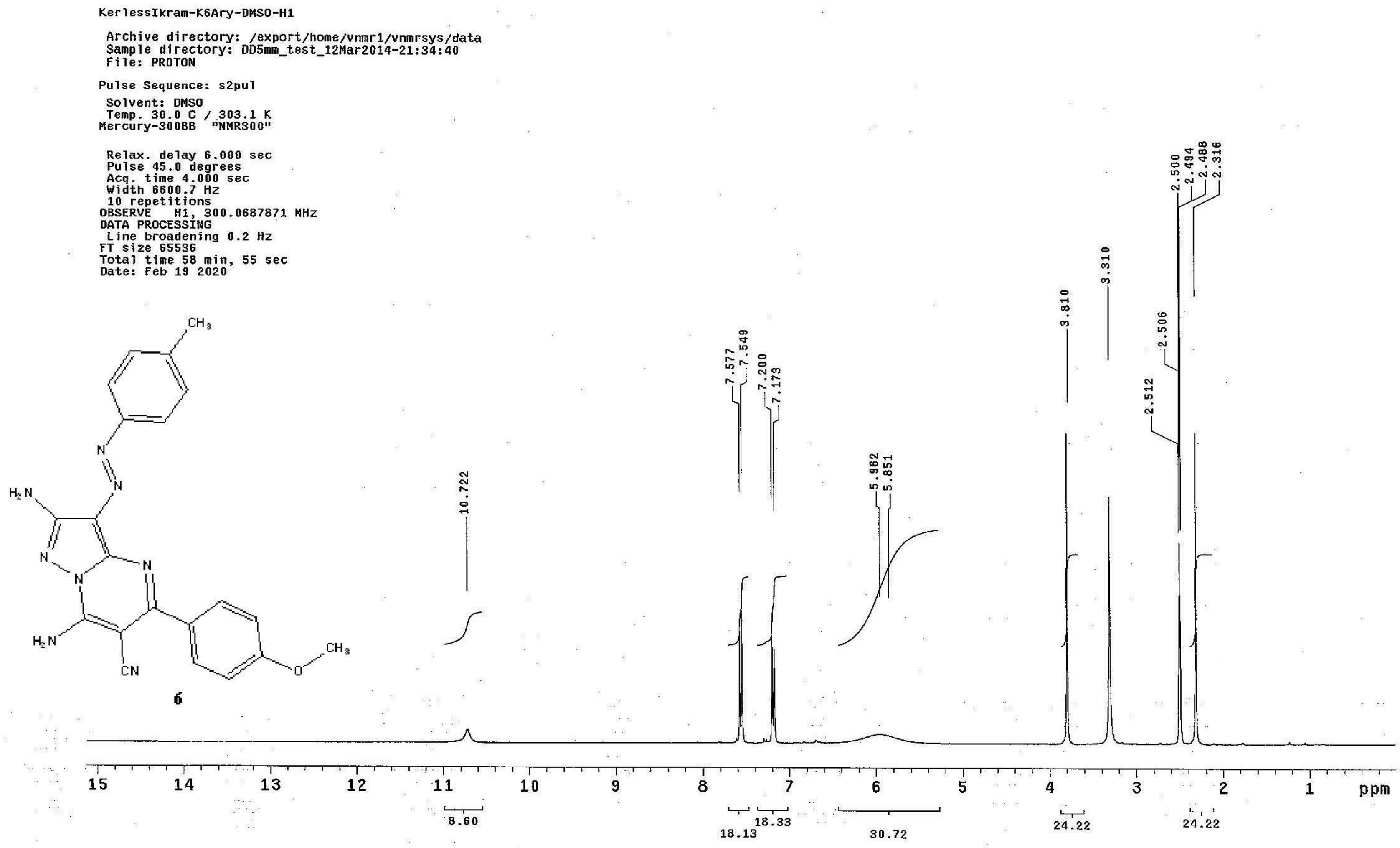

1H-NMR-D2O spectrum of compound 6

KerlexxIkram-K6Ary-DMSO-D2O-H1

Archive directory: /export/home/vnmr1/vnmrsys/data
Sample directory: DD5mm_test_12Mar2014-21:34:40
File: PROTON

Pulse Sequence: s2pul

Solvent: DMSO
Temp. 30.0 C / 303.1 K
Mercury-300BB "NMR300"

Relax. delay 6.000 sec
Pulse 45.0 degrees
Acq. time 4.000 sec
Width 6600.7 Hz
14 repetitions
OBSERVE H1, 300.0687871 MHz
DATA PROCESSING
Line broadening 0.2 Hz
FT size 65536
Total time 58 min, 55 sec
Date: Feb 19 2020

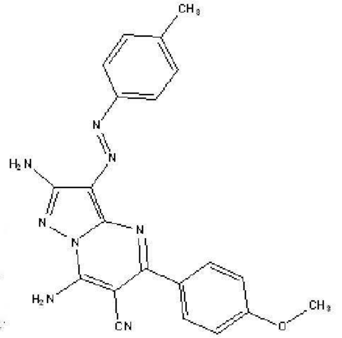

6

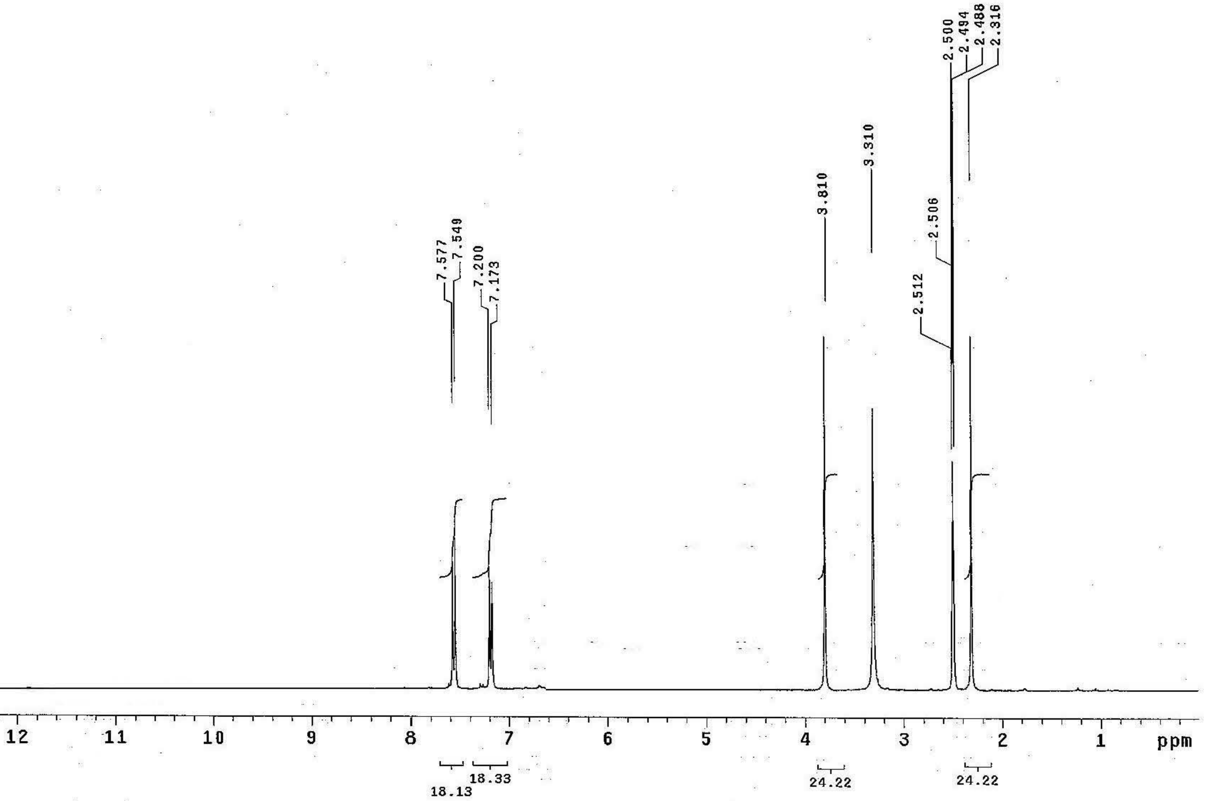

13C-NMR spectrum of compound 6

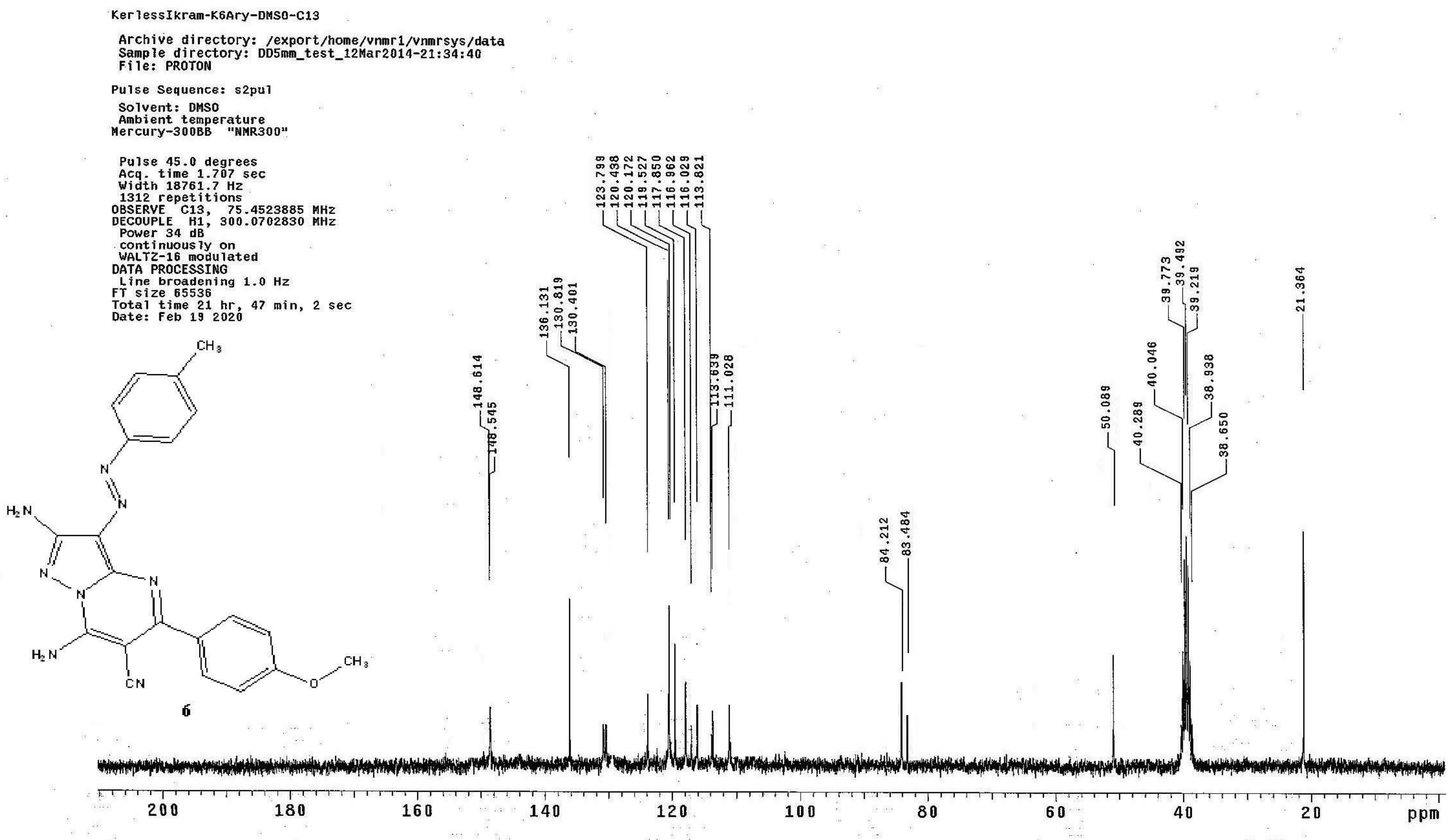

mass spectrum of compound 6

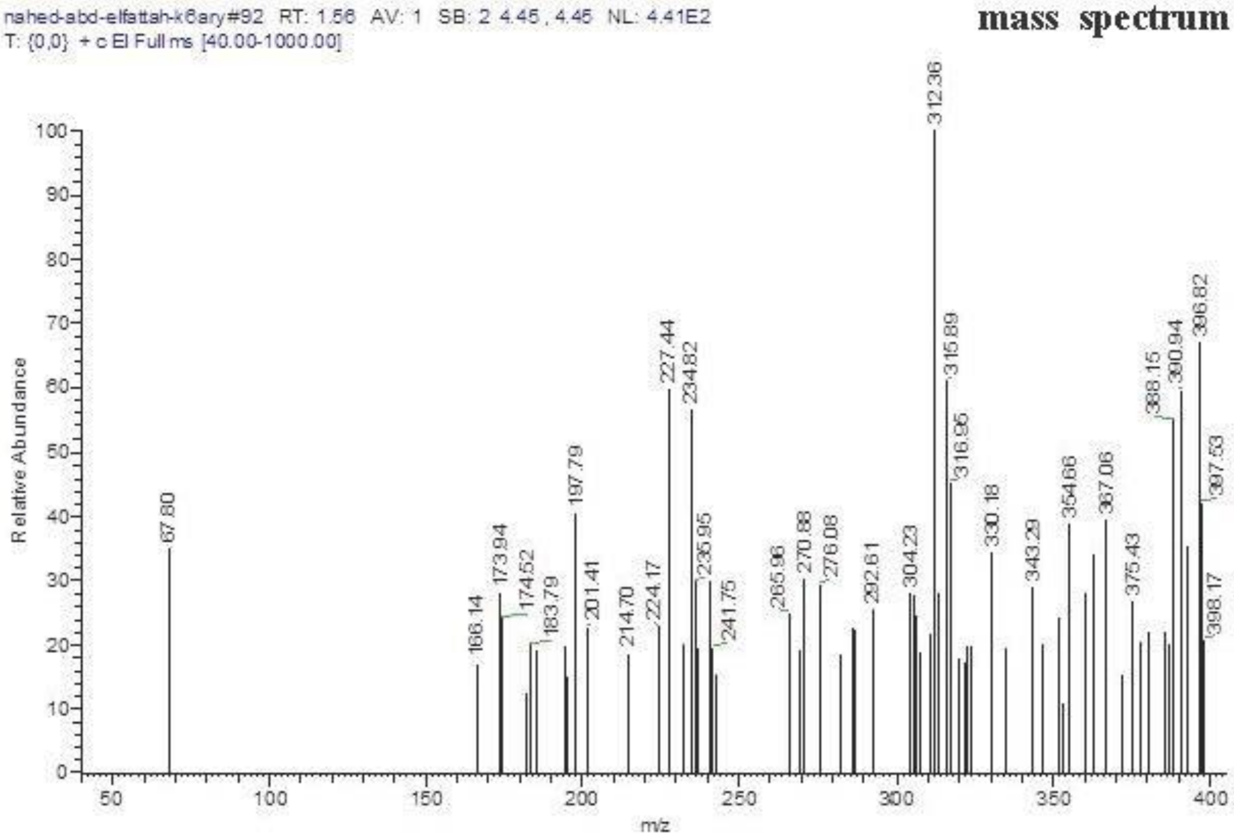

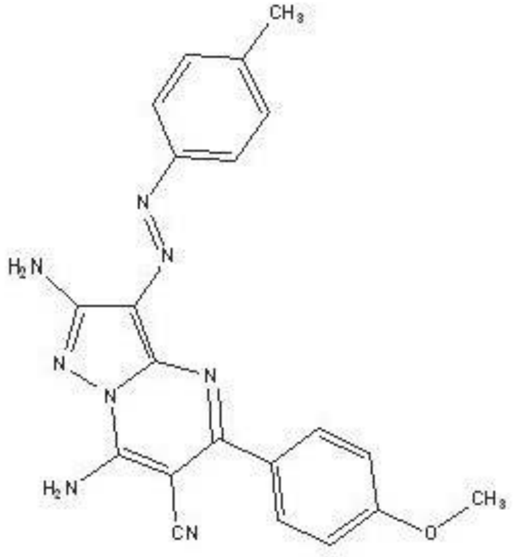

6

K6Mn.0

IR spectrum of compound 7

12/2/2019

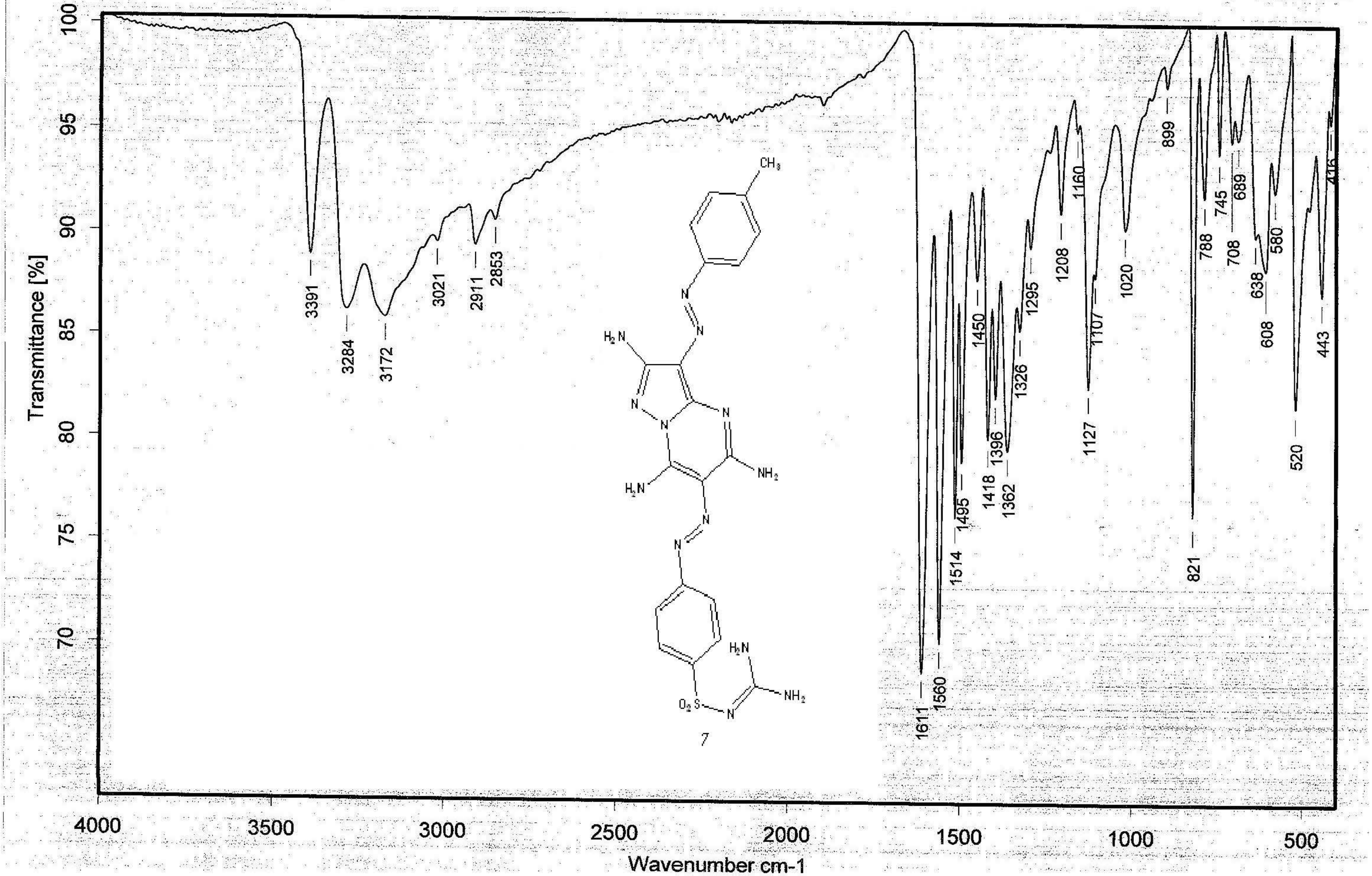

1H-NMR-D2O spectrum of compound 7

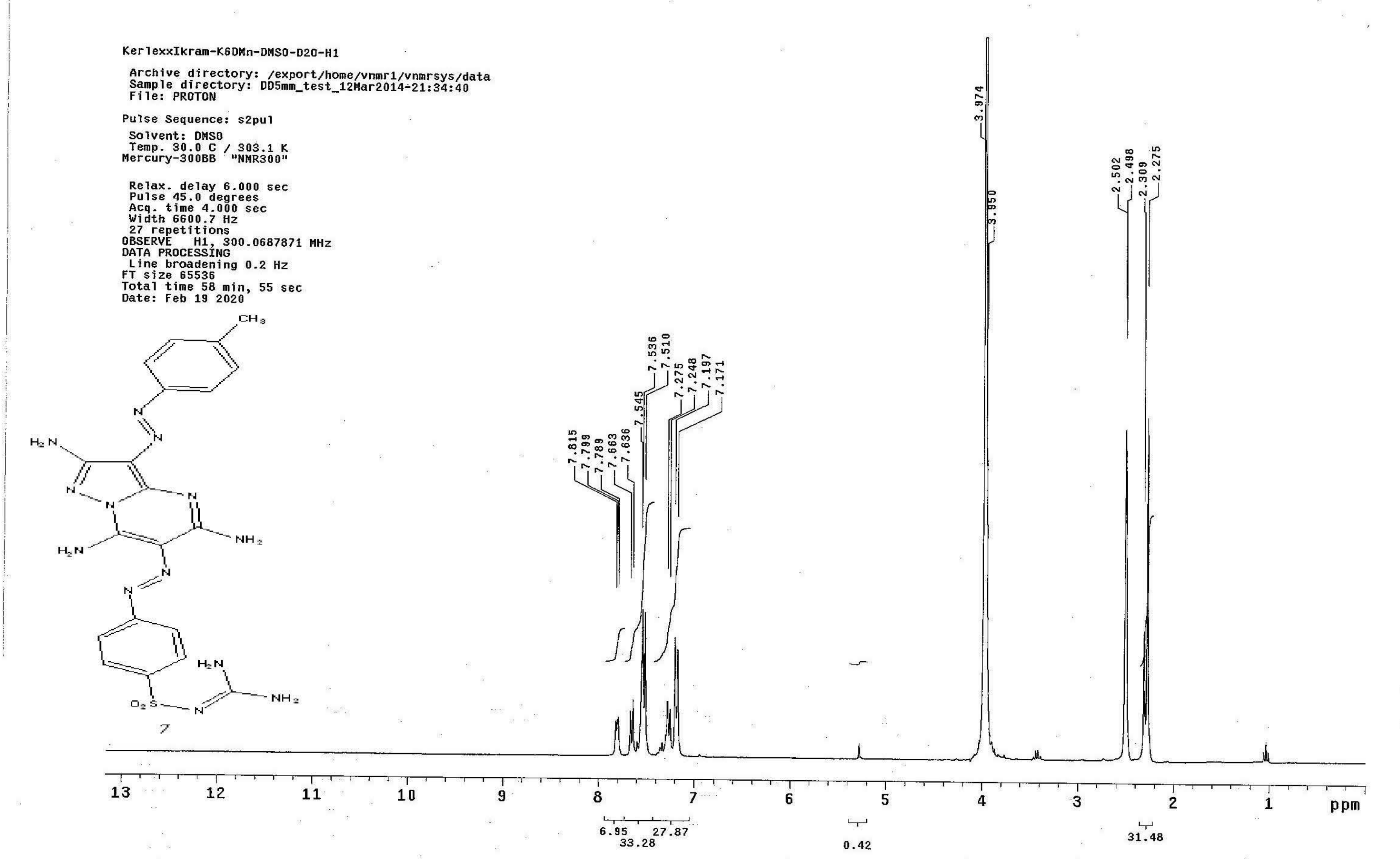

1H-NMR spectrum of compound 7

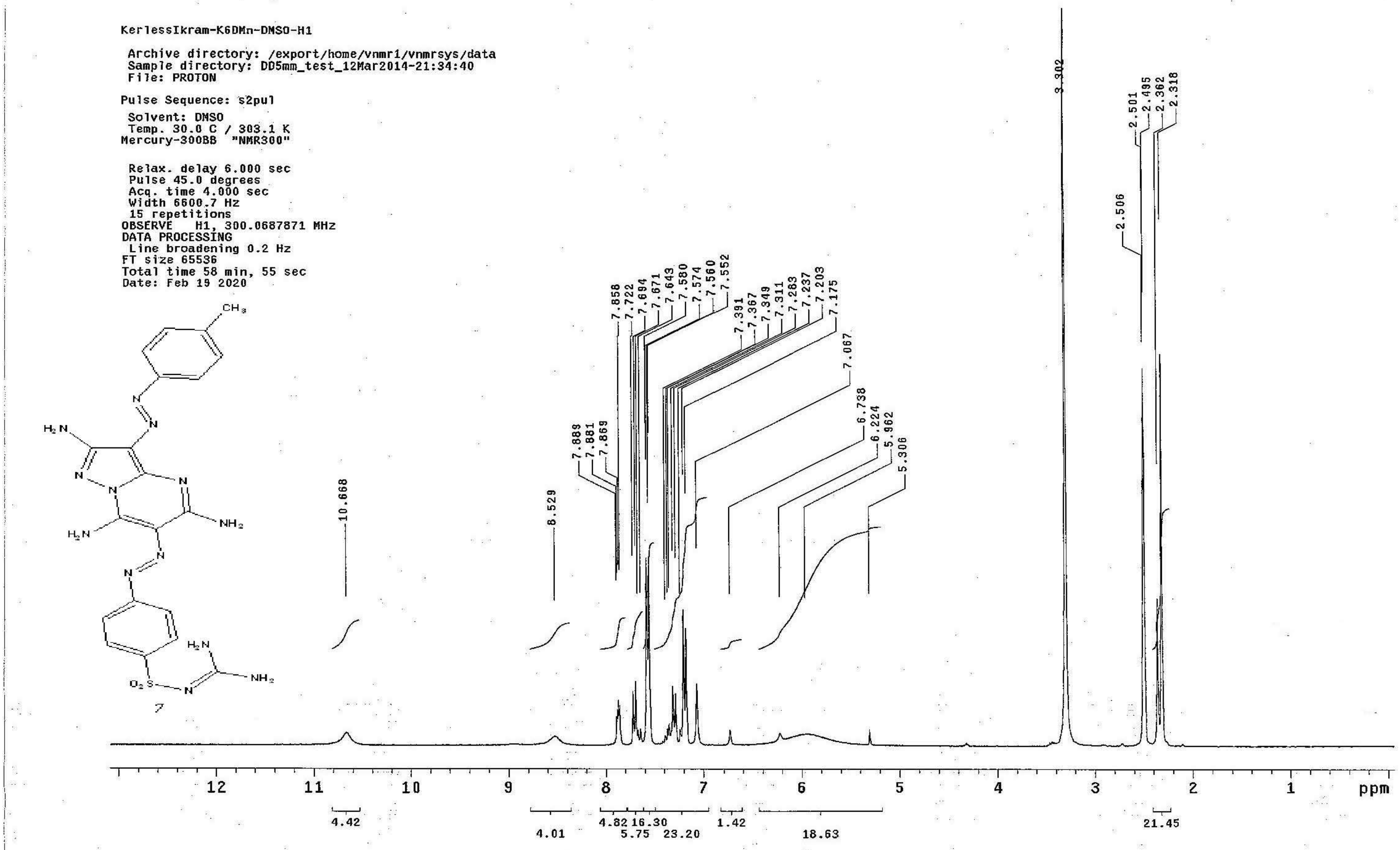

mass spectrum of compound 7

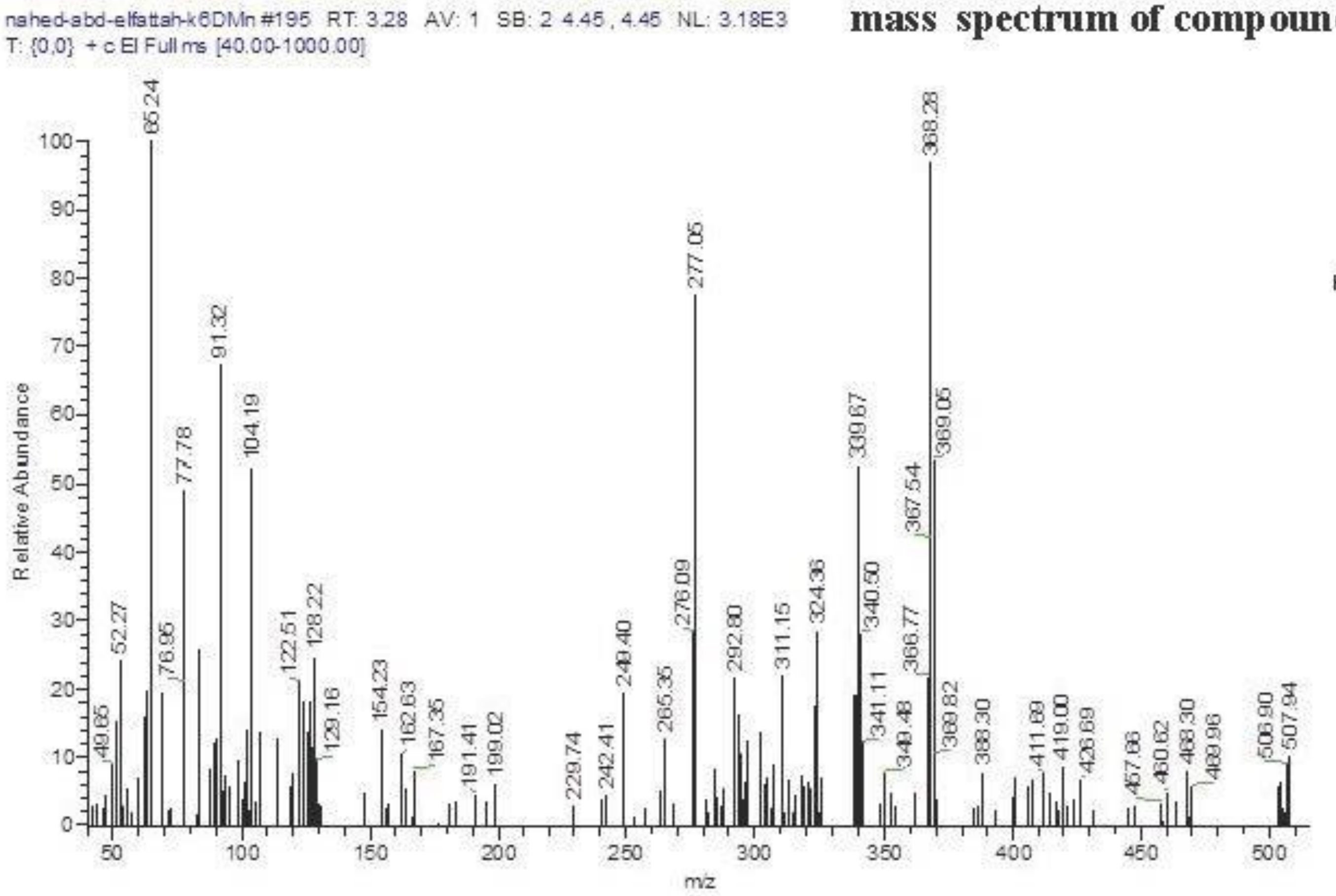

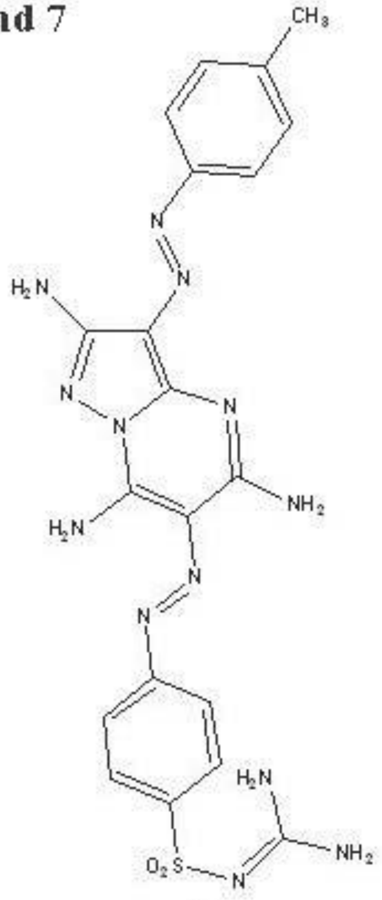

7
